# Association between Homologous Recombination Repair Defect Status and Long-Term Prognosis of Early HER2-Low Breast Cancer: A Retrospective Cohort Study

**DOI:** 10.1093/oncolo/oyae021

**Published:** 2024-02-16

**Authors:** Jiayi Chen, Yingying Zhu, Wei Wu, Yaqi Xu, Wenqian Yang, Li Ling, Qun Lin, Shijie Jia, Yuan Xia, Zihao Liu, Yaping Yang, Chang Gong

**Affiliations:** Guangdong Provincial Key Laboratory of Malignant Tumor Epigenetics and Gene Regulation, Sun Yat-Sen Memorial Hospital, Sun Yat-Sen University, Guangzhou, People’s Republic of China; Department of Breast Surgery, Breast Tumor Center, Sun Yat-Sen Memorial Hospital, Sun Yat-Sen University, Guangzhou, People’s Republic of China; Division of Clinical Research Design, Clinical Research Center, Sun Yat-Sen Memorial Hospital, Sun Yat-Sen University, Guangzhou, People’s Republic of China; Guangdong Provincial Key Laboratory of Malignant Tumor Epigenetics and Gene Regulation, Sun Yat-Sen Memorial Hospital, Sun Yat-Sen University, Guangzhou, People’s Republic of China; Department of Breast Surgery, Breast Tumor Center, Sun Yat-Sen Memorial Hospital, Sun Yat-Sen University, Guangzhou, People’s Republic of China; Guangdong Provincial Key Laboratory of Malignant Tumor Epigenetics and Gene Regulation, Sun Yat-Sen Memorial Hospital, Sun Yat-Sen University, Guangzhou, People’s Republic of China; Department of Breast Surgery, Breast Tumor Center, Sun Yat-Sen Memorial Hospital, Sun Yat-Sen University, Guangzhou, People’s Republic of China; Guangdong Provincial Key Laboratory of Malignant Tumor Epigenetics and Gene Regulation, Sun Yat-Sen Memorial Hospital, Sun Yat-Sen University, Guangzhou, People’s Republic of China; Department of Breast Surgery, Breast Tumor Center, Sun Yat-Sen Memorial Hospital, Sun Yat-Sen University, Guangzhou, People’s Republic of China; Department of Medical Statistics, School of Public Health, Sun Yat-Sen University, Guangzhou, China; Guangdong Provincial Key Laboratory of Malignant Tumor Epigenetics and Gene Regulation, Sun Yat-Sen Memorial Hospital, Sun Yat-Sen University, Guangzhou, People’s Republic of China; Department of Breast Surgery, Breast Tumor Center, Sun Yat-Sen Memorial Hospital, Sun Yat-Sen University, Guangzhou, People’s Republic of China; Guangdong Provincial Key Laboratory of Malignant Tumor Epigenetics and Gene Regulation, Sun Yat-Sen Memorial Hospital, Sun Yat-Sen University, Guangzhou, People’s Republic of China; Department of Breast Surgery, Breast Tumor Center, Sun Yat-Sen Memorial Hospital, Sun Yat-Sen University, Guangzhou, People’s Republic of China; Guangdong Provincial Key Laboratory of Malignant Tumor Epigenetics and Gene Regulation, Sun Yat-Sen Memorial Hospital, Sun Yat-Sen University, Guangzhou, People’s Republic of China; Department of Breast Surgery, Breast Tumor Center, Sun Yat-Sen Memorial Hospital, Sun Yat-Sen University, Guangzhou, People’s Republic of China; Department of Breast Surgery, Department of General Surgery, Shenzhen People’s Hospital, The Second Clinical Medical College of Jinan University, the First Affiliated Hospital of Southern University of Science and Technology, Shenzhen, Guangdong, People’s Republic of China; Guangdong Provincial Key Laboratory of Malignant Tumor Epigenetics and Gene Regulation, Sun Yat-Sen Memorial Hospital, Sun Yat-Sen University, Guangzhou, People’s Republic of China; Department of Breast Surgery, Breast Tumor Center, Sun Yat-Sen Memorial Hospital, Sun Yat-Sen University, Guangzhou, People’s Republic of China; Guangdong Provincial Key Laboratory of Malignant Tumor Epigenetics and Gene Regulation, Sun Yat-Sen Memorial Hospital, Sun Yat-Sen University, Guangzhou, People’s Republic of China; Department of Breast Surgery, Breast Tumor Center, Sun Yat-Sen Memorial Hospital, Sun Yat-Sen University, Guangzhou, People’s Republic of China

**Keywords:** HER2-low, early breast cancer, homologous recombination deficiency, TCGA

## Abstract

**Background:**

As a newly identified subtype of HER2-negative tumors associated with a less favorable prognosis, it remains crucial to evaluate potential prognostic and predictive factors, particularly non-invasive biomarkers, for individuals with human epidermal growth factor 2 (HER2) low early-stage breast cancer (EBC). Multiple investigations have highlighted that HER2-negative patients with EBC exhibiting high homologous recombination deficiency (HRD) scores display lower rates of pathological complete response (PCR) to neoadjuvant chemotherapy (NAC). Nevertheless, no study to date has explored the correlation between HRD and the long-term prognosis in HER2-low patients with EBC.

**Patients and methods:**

This retrospective observational study focuses on primary EBC sourced from The Cancer Genome Atlas dataset (TCGA). It reveals the gene mutation landscape in EBC with low HER2 expression and elucidates the tumor immune landscape across different HRD states. Utilizing bioinformatics analysis and Cox proportional models, along with the Kaplan-Meier method, the study assesses the correlation between HRD status and disease-specific survival (DSS), disease-free interval (DFI), and progression-free interval (PFI). Subgroup analyses were conducted to identify potential variations in the association between HRD and prognosis.

**Results:**

In the patients with HER2-low breast cancer, patients with homologous recombination related genes (HRRGs) defects had an HRD score about twice that of those without related genes mutations, and were at higher risk of acquiring *ARID1A*, *ATM*, and *BRCA2* mutations. We also found that most immune cell abundances were significantly higher in EBC tumors with high HRD than in EBC tumors with low HRD or HRD-medium, particularly plasma B-cell abundance, CD8 T-cell abundance, and M1 macrophages. In addition, these tumors with HRD-high also appear to have significantly higher tumor immune scores and lower interstitial scores. Then, we analyzed the relationship between different HRD status and prognosis. There was statistical significance (*P* = .036 and *P* = .046, respectively) in DSS and PFI between the HRD-low and HRD-high groups, and patients with HRD-high EBC showed relatively poor survival outcomes. A medium HRD score (hazard ratio, HR = 2.15, 95% CI: 1.04-4.41, *P *= .038) was a significant risk factor for PFI. Hormone receptor positivity is an important factor in obtaining medium-high HRD score and poor prognosis.

**Conclusion:**

Higher HRD scores were associated with poorer PFI outcomes, particularly in people with HR+/HER2-low. Varied HRD states exhibited distinctions in HRRGs and the tumor immune landscape. These insights have the potential to assist clinicians in promptly identifying high-risk groups and tailoring personalized treatments for patients with HER2-low EBC, aiming to enhance long-term outcomes.

## Teaser

This study explored the associations between homologous recombination deficiency status and the risk of long-term prognosis of patients with HER2-low early-stage breast cancer to guide treatment decisions.

**Implications for Practice**

In this study, we selected HRD score as a novel biomarker to demonstrate the outcomes of early breast cancer (EBC) patients with low HER2 status. High HRD scores predict poor survival in patients with EBC, especially in patients with positive hormone receptors, and these patients need special attention and active treatment. Accurate assessment of HRD status in clinical practice enables physicians to choose appropriate treatment plans and improves patient survival. This provides crucial evidence for personalized therapy and highlights the need for more proactive treatment strategies for patients with high HRD scores.

## Introduction

Approximately 70% of early-stage breast cancers (EBC) are human epidermal growth factor 2 (HER2) negative.^1^ Importantly, the characterization of HER2 has dramatically evolved over the last 3 decades, from a poor prognostic biomarker to a predictor of clinical benefits of anti-HER2 therapy.^2,3^ Strikingly, patients who are defined as HER2-low based on the 2018 American Society of Clinical Oncology/College of American Pathologists (ASCO/CAP)^4^ guidelines can benefit from novel anti-HER2 antibody-conjugated (ADC) therapy. Conventional HER2 negative status, which is defined as immunohistochemistry (IHC) 0, 1+, 2+, and fluorescence in situ hybridization (FISH) negative status, can be divided into HER2-0 (IHC 0) and HER2-low (IHC 1+/FISH negative or IHC 2+/FISH negative) based on the ASCO/CAP criteria. More than 50% of breast tumors that had HER2-negative status were actually HER2-low EBC.^5-11^ Compared with HER2-0 expression, patients with HER2-low EBC had significantly lower rates of pathological complete response (PCR) to neoadjuvant chemotherapy (NAC) and had poorer overall survival (OS).This may be attributed to the fact that patients with HER2-low EBC often had large primary tumors, axillary lymph node involvement, higher histopathological grade, and expression levels of the tumor proliferation gene Ki-67 at the first consultancy.^12^ Therefore, it is still imperative to identify potential predictive factors, especially noninvasive biomarkers, for the prognosis of patients with HER2-low EBC to improve their survival.

Homologous recombination repair (HRR), the preferred mechanism for repairing double-strand breaks (DSBs) in DNA, has attracted the attention of academia in recent years.^13,14^ Homologous recombination deficiency (HRD), which is caused by HRR dysfunction, often results in impaired genome integrity, reduced fidelity of genetic information transmission, and increased breast tumorigenesis.^15-17^ Several studies have reported that patients with HER2-negative EBC with low HRD score shad a high NAC PCR rate.^18-21^ However, no study has been conducted to evaluate the relationship between HRD and long-term prognosis in patients with HER2-low EBC. Thus, we performed a comprehensive analysis to explore the associations between HRD status and the risk of long-term prognosis of patients with HER2-low EBC to guide physicians’ treatment decisions.

## Materials and Methods

### Inclusion Criteria, Exclusion Criteria, Data Collection and Processing

Patient with breast cancer demographics, tumor characteristics, corresponding clinical follow-up information and Gene counts, and the relative expression matrix were originated from The Cancer Genome Atlas (TCGA) database (https://portal.gdc.cancer.gov) and UCSC Xena (https://xenabrowser.net). The controlled TCGA data were approved by the TCGA committee. In total, 1080 TCGA-BRCA sample data points were extracted. The main inclusion criteria in this study were patients with HER2-negative breast cancer diagnosed with stages I-III disease, followed by complete information on age, tumor size, lymph node metastasis, hormone receptor status, and survival prognosis. Patients with positive (IHC 3 + or FISH positive) HER2 or unavailable HER2 status, age < 18 years, distant metastases, or lack of follow-up information were excluded. Finally, 823 patients (531 HER2-low and 292 HER2-0) from TCGA who met the inclusion and exclusion criteria were included in our study. In addition, we collected Sun Yat-sen memorial hospital (SYSU) and Fudan University Shanghai Cancer Center (FUSCC) datasets for verification. SYSU dataset was approved by the Institutional Review Board of SYSU (IRB ID: ChiCTR2200061861). All enrolled patients were fully informed of their rights and signed written consent forms. One hundred and ten patients diagnosed with HER2-low early breast cancer (EBC) who were treated at the Department of Breast Surgery at SYSU from January 1, 2022, to November, 2023, were retrospectively enrolled in this study. FUSCC datasets were obtained from the Genome Sequence Archive (GSA) under accession number PRJCA017539 and the supplementary files of the published study.^22^

Definition of clinical information of breast cancer extracted clinical information included patient demographics, tumor characteristics including estrogen receptor (ER), progesterone receptor (PR), HER2 IHC and HER2 FISH status based on the prevailing ASCO-CAP recommendations,^23-26^ and tumor stage (TNM), tumor size (T), and lymph node metastasis status (LN) classification by the sixth edition American Joint Committee on Cancer (AJCC).^27^ HER2 status was evaluated according to the “HER2 IHC score,” “HER2 IHC status,” and “fluorescence in situ hybridization (FISH) status” available in the TCGA dataset. Patients with an HER2 IHC score of 2+ were included only if their HER2 status by FISH was available and not defined as equivocal. Patients with HER2 IHC scores “not available” were included in the analysis only if HER2 IHC status was available. Among them, HER2-low patients were defined as having an IHC score of 1+ or 2+ without HER2 FISH amplification.^28^ER and PR status were characterized according to the IHC data available, and ER and PR positivity were defined as having >1% tumor nucleus staining. Hormone receptor (HR) positivity (HR+) was defined as ER and/or PR status positivity.^29^

### HRD Score Assessment

HRD scores were defined as the unweighted sum of the scores for 3 ultrastructural features: large-scale state transitions (LST), telomere allelic imbalances (TAI), and loss-of-heterozygosity (LOH).^30,31^ LOH was defined as the number of counts of chromosomal LOH regions shorter than the whole chromosome and longer than 15 Mb.^32^ LST were defined as chromosome breakpoints (change in copy number or allele content) between adjacent regions each of at least 10 Mb obtained after smoothing and filtering shorter than 3 Mb small-scale copy number variation.^33^ TAI was defined as the number of regions with allele imbalance that extended to the sub telomere but did not cross the centromere.^34^

### Identification of the HRR Gene Mutation Panel

The key HRR gene (HRRGs) panel includes genes associated with a common homologous recombination repair pathway. Genes such as AT-rich interaction domain 1A (*ARID1A*), ataxia telangiectasia-mutated (*ATM*), ATM and Rad3 related (*ATR*), alpha thalassemia retardation syndrome X-linked (*ATRX*), BRCA1-associated protein 1 (*BAP1*), BRCA1-associated ring domain (*BARD1*), Bloom syndrome helicase (*BLM*), breast cancer susceptibility gene 1/2 (*BRCA1/2*), cyclin-dependent kinases 12 (*CDK12*), checkpoint kinase 1/2 (*CHEK1/2*), Fanconi anemia complementation group (*FANCA/C/D2/F/G/I/M*), meiotic recombination 11 homolog A (*MRE11A*), Nibrin (*NBN*), partner and localizer of BRCA2 (*PALB2*), RAD50 double-strand break repair protein (*RAD50*), RAD51 recombinase B/C (*RAD51B/C*), RAD54 Like (*RAD54L*), replicating protein A1 (*RPA1*), and Wemer syndrome protein (*WRN*) were included in this study.^35^

### Immune Infiltration Analysis

The relative expression matrix, which was downloaded from the TCGA database, was used to calculate the absolute infiltration score of tumor samples by CIBERSORT-ABS^36^ and XCELL.^37^ Each immune cell fraction was merged through the corresponding TCGA ID number. We compared the expression levels of multiple tumor-infiltrating immune cells (TIICs) between 3 HRD states using the Wilcoxon tests and drew boxplots to unveil the results of XCELL and CIBERSORT-ABS analyses to predict the potential impact of immunotherapy based on HRD status.

### Outcome Measures

Survival data, including disease-specific survival (DSS), disease-free interval (DFI), and progression-free interval (PFI), were obtained from the TCGA Pan-Cancer Clinical Data Resource (TCGA-CDR).^38^ All prognostic indicators included in this paper are based on the American Food and Drug Administration criteria. DSS was defined as the percent of people who die from breast cancer. DFI was defined as the time from diagnosis until the date of the first new tumor event subsequent to the determination of a patient’s disease-free status after their initial diagnosis and treatment. The PFI refers to the time from diagnosis until the date of first occurrence of a new tumor event, which included disease progression, locoregional recurrence, distant metastasis, new primary tumor, or death with tumor.^2,38^ OS was defined as the time from the initial surgery to the date of death due to any cause or the last follow-up date in the FUSCC dataset.

### Statistical Analysis

Baseline characteristics of all patients were represented as frequencies and percentages for categorical variables. The χ^2^ test or Fisher’s exact test was performed to detect differences in characteristics among the HRD-low, HRD-medium, and HRD-high groups. Unpaired *t* test was used to analyze the differences in clinical features and mutation status in HRD scores. Wilcoxon signed-rank test was used for immune cell fraction comparison. A 2-sided *P-*value < .05 was considered statistically significant. All statistical analyses and visualization were performed with SPSS version 25.0 (SPSS Inc.), GraphPad Prism software 9.0.0 (GraphPad Software, San Diego, California USA), or R package. Survival analyses were performed using the Kaplan-Meier method, and the log-rank test was used to compare the statistical significance between patients with HER2-low EBC with different HRD scores. The hazard ratio (HR) and 95% CI were determined through a Cox proportional hazards regression model to test the relations between different HRD states and DSS, DFI, and PFI. A statistical significance level of .05 was used to select variables for inclusion in multivariate regression analysis. Patients with breast cancer were sorted in ascending order of HRD scores according to the interquartile range. The patients were assigned into 3 groups: HRD-low (HRD score ≤ 8), HRD-medium (8 < HRD score < 33), and HRD-high (HRD score ≥ 33).

## Results

### Clinical Characteristics of HER2-Low EBC Patients in the TCGA Cohort

A total of 1080 patients with breast cancer were enrolled from TCGA, of whom 184 were excluded because of non-early-stage breast cancer. In addition, 66 patients who were HER2-positive or unavailable HER2 status, and 3 patients who lacked complete survival or clinicopathological data were excluded. Ultimately, 531 HER2-low and 292 HER2-0 EBC patients were enrolled in the study and the screening strategy flow chart is shown in Supplementary Fig. S1. In all, 531 patients with HER2-low EBC, 140 (26.4%) patients were classified into the HRD-low group, 262 (49.3%) patients were in the HRD-medium group, and 129 (24.3%) patients were classified into the HRD-high group based on the cutoff value mentioned in Methods section. The clinical characteristics of the 3 different groups of HER2-low EBC are presented in Table 1. The majority of patients were age < 60 (52.7%), stages I-II (72.3%), cT1-T2 (83.1%), cN0-N1 (81.4%), IHC 1 + (62.5%), and hormone receptor positive (82.9%). Most patients did not carry homologous recombination-related gene mutations (61.2%) or BRCA1/2 (73.1%) mutations. There were statistically significant differences in age, tumor size, lymph node status, hormone receptor status, somatic BRCA1/2, and other homologous recombination-related gene mutations in the 3 HRD score groups at baseline (*P *< .05). The baseline pathological characteristics of SYSU-EBC and FUSCC-EBC were presented in Supplementary Table S1. In all, 110 patients with HER2-low EBC from SYSU and 270 HR+ patients with HER2-low EBC from FUSCC were enrolled in the study. The majority of SYSU patients were age < 60 (88.18%), stages I-II (65.45%), cT1-T2 (70.91%), cN0-N1 (78.18%), IHC 2 + (58.18%), and hormone receptor positive (92.73%), and the majority of patients with FUSCC were age < 60 (74.44%), stages I-II (67.04%), cT1-T2 (99.63%), cN0-N1 (71.11%), and IHC 1 + (59.63%). Considering that whether HRD score associates with HER2 status or not, we also explore their relationships in the TCGA HER2-0 EBC cohort. We did not find any significant differences in the clinicopathological characteristics of HER2-0 EBC, but there were still significant differences in homologous recombination-related gene mutations (*P* = .011; Supplementary Table S2).

**Table 1.** Clinical baseline characteristics among different status of the HRD score in HER2-low TCGA-EBC.

TCGA HER2-low clinical characteristic	All patients (*n* = 531)	Number of patients (%)			
		HRD ≤ 8 (*n* = 140)	HRD 9-33 (*n* = 262)	HRD > 33 (*n* = 129)	*P*-value
Age (years)					<0.001
< 60	280 (52.73%)	52 (37.14%)	145 (55.34%)	83 (64.34%)	
≥ 60	251 (47.27%)	88 (62.86%)	117 (44.66%)	46 (35.66%)	
TNM stage					0.111
Stages I-II	384 (72.32%)	94 (67.14%)	200 (76.34%)	90 (69.77%)	
Stage III	147 (27.68%)	46 (32.86%)	62 (23.66%)	39 (30.23%)	
Tumor size					<0.001
T1-T2	441 (83.05%)	98 (70.00%)	225 (85.88%)	118 (91.47%)	
T3-T4	90 (16.95%)	42 (30.00%)	37 (14.12%)	11 (8.53%)	
Lymph nodes					0.003
N0-N1	432 (81.36%)	123 (87.86%)	216 (82.48%)	93 (72.09%)	
N2-N3	99 (18.64%)	17 (12.14%)	46 (17.56%)	36 (27.91%)	
IHC					0.577
1+	332 (62.52%)	91 (65.00%)	158 (60.31%)	83 (64.34%)	
2+	199 (37.48%)	49 (35.00%)	104 (39.69%)	46 (35.66%)	
HR status					<0.001
Negative	91 (17.14%)	4 (2.86%)	30 (11.45%)	57 (44.19%)	
Positive	440 (82.86%)	136 (97.14%)	232 (88.55%)	72 (55.81%)	
HRRGs mutations status					0.001
NO	325 (61.21%)	98 (70.00%)	164 (62.6%)	63 (48.84%)	
YES	80 (15.07%)	12 (8.57%)	35 (13.36%)	33 (25.58%)	
NA	126 (23.73%)	30 (21.43%)	63 (24.05%)	33 (25.58%)	
BRCA1/2 mutation status					0.015
NO	388 (73.07%)	109 (77.86%)	193 (73.66%)	86 (66.67%)	
YES	17 (3.20%)	1 (0.71%)	6 (2.29%)	10 (7.75%)	
NA	126 (23.73%)	30 (21.43%)	63 (24.05%)	33 (25.58%)	

Abbreviations: BRCA, breast cancer susceptibility gene; HR, hormone receptor; HRD, homologous recombination defect score; HRRGs, homologous recombination repair genes; IHC, immunohistochemistry; TCGA, The Cancer Genome Atlas dataset.

### HRD Score Distribution and Genetic Mutation Landscape of HRRGs in Patients With HER2-Low EBC

**Figure 1.**
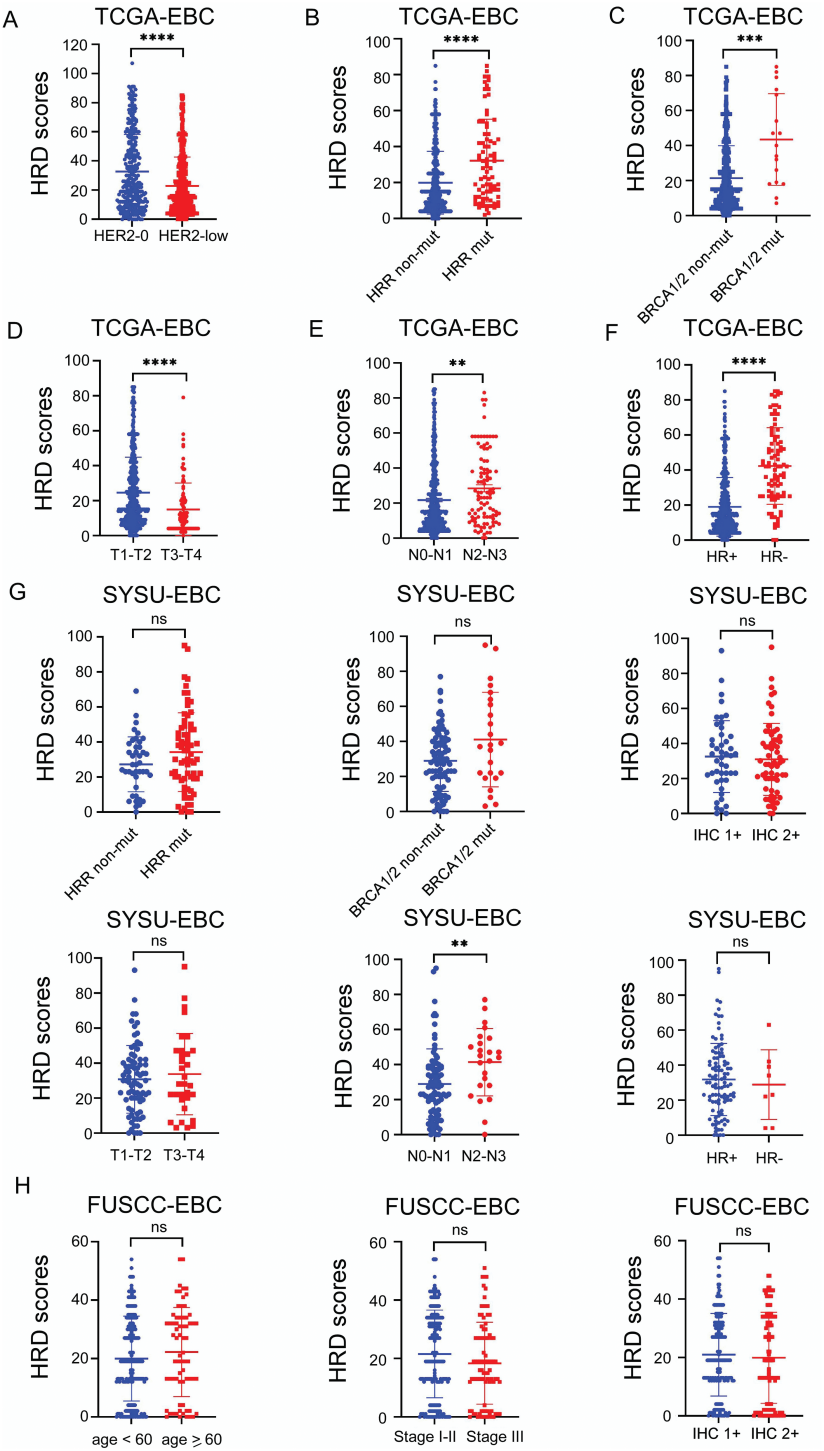
Association between HRD score and patients with HER2-low early-stage breast cancer scatter diagram depicting the HRD score expression in HRR-related genes and various clinical features. (Wilcoxon signed-rank test, *P and **P < .05, ***P = .001, ****P < .001).

First, we compared the mean HRD score of patients with HER2-0 and HER2-low TCGA-EBC, and the mean HRD score of patients with HER2-0 was significantly higher than that of patients with HER2-low (32.77 vs 22.98, *P *< .0001, Fig. 1A). Analyses of 80 HRRGs mutation and 325 non-HRRG mutation breast samples indicated that the mean HRD score was 32.24 in HRRG mutation-carrier patients, while the mean HRD score was 19.90 (*P *< .0001) in non-HRRG mutation-carrier patients (Fig. 1B). Similar results were observed in the analysis of breast samples with or without *BRCA1/2* mutations, and the mean HRD scores were 2-fold higher in *BRCA1/2* mutation-carrying patients than in non-*BRCA1/2* mutation-carrying patients (43.47 vs 21.41, *P* = .0001, Fig. 1C). Further analysis revealed that the HRD score was significantly elevated in cT1-T2, cN2-N3, and patients with HR-negative EBC (Fig. 1D-F). In addition, we further compared the HRD scores of all subgroups in our validation cohort. Consistently, we found that the mean HRD score of patients with N2-N3 was significantly higher than those with N0-N1 (41.33 vs 28.87, *P* = .0024, Fig. 1G) in SYSU-EBC. However, no statistically significant differences were observed in other subgroups (Fig. 1G-H). Next, we explored somatic mutations of the top 28 HRRGs in HER2-low TCGA-EBC and SYSU-EBC. As depicted in Supplementary Fig. S2A, 137 of 531 (25.80%) HER2-low EBC samples presented somatic genetic mutations, and the findings suggested *ARID1A* had the highest mutation incidence, followed by *ATM* and *BRCA2* among the 28 HRRGs. In the HER2-low SYSU-EBC, there was a high incidence of somatic mutations in BRCA2, followed by NBN, PALB2, and BRCA1 (Supplementary Fig. S2B).

### Immunological Tumor Landscape of HER2-Low EBCs According to HRD Status

**Figure 2.**
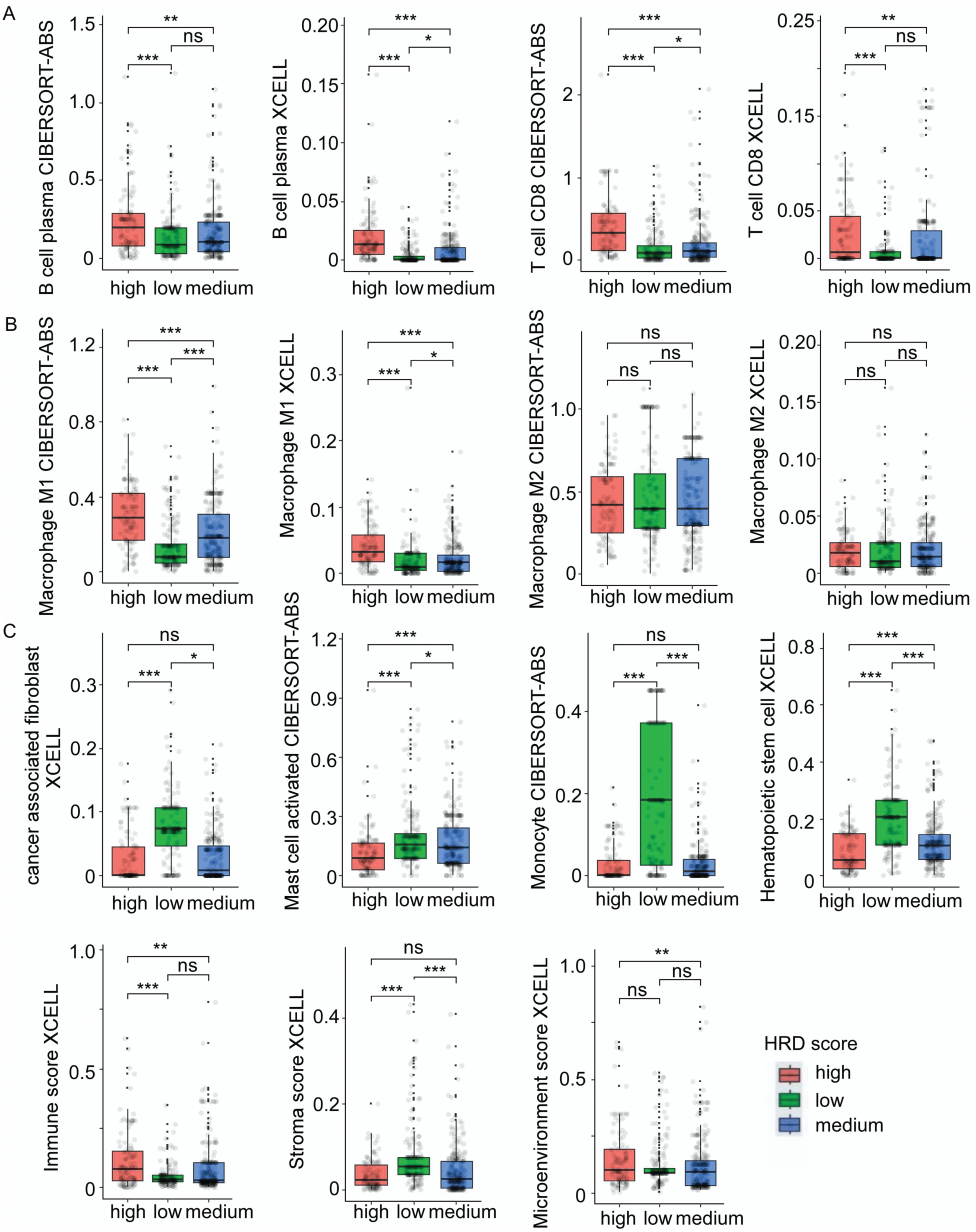
Immunological tumor landscape of HER2-low TCGA-EBC according to HRD status. Box plots representing the distribution of B-cell and CD8 T-cell abundance (**A**), macrophages subpopulations (**B**), and other immune signatures (**C**) according to HRD status in HER2-low tumors (Wilcoxon-signed rank test, *P < .05, **P < .01, ***P < .001).

To explore the immune microenvironment between different HRD states, we used 2 algorithms, XCELL and CIBERSORT-ABS, to analyze the immunological landscape of 3 different HRD HER2-low EBC groups (Supplementary Fig. S3). In our analysis, there are significant differences between 3 different HRD groups, most immune cell abundances were significantly higher in HRD-high EBC tumors than in HRD-low or HRD-medium EBC tumors, notably plasma B-cell abundance and CD8+ T-cell abundance (Fig. 2A). On the contrary, activated mast cells, monocyte, cancer-associated fibroblast (CAF), and hematopoietic stem cell (HSC), were all significantly higher in HRD-low compared to HRD-high tumors (Fig. 2B). Concerning macrophages and NK-cell subpopulations, macrophage M1 were found to be higher in HRD-high EBC tumors when compared to HRD-low and HRD-medium cases, but no significant difference was observed in terms of macrophage M2 (Fig. 2C) and parameters associated with NK-cell lineage (not shown). Moreover, these HRD-high tumors also appeared to have significantly higher tumor immune scores, and lower stroma score than HRD-low tumors (Fig. 2C). Collectively, these results therefore show that different immune cell landscapes exist between different HRD states in HER2-low EBC tumors, and apparently more favorable to therapeutic approaches using immune checkpoint blockers, compared to HRD-low tumors.

### High HRD Score Predicts Poor Prognosis of Patients With HER2-Low EBC

**Figure 3.**
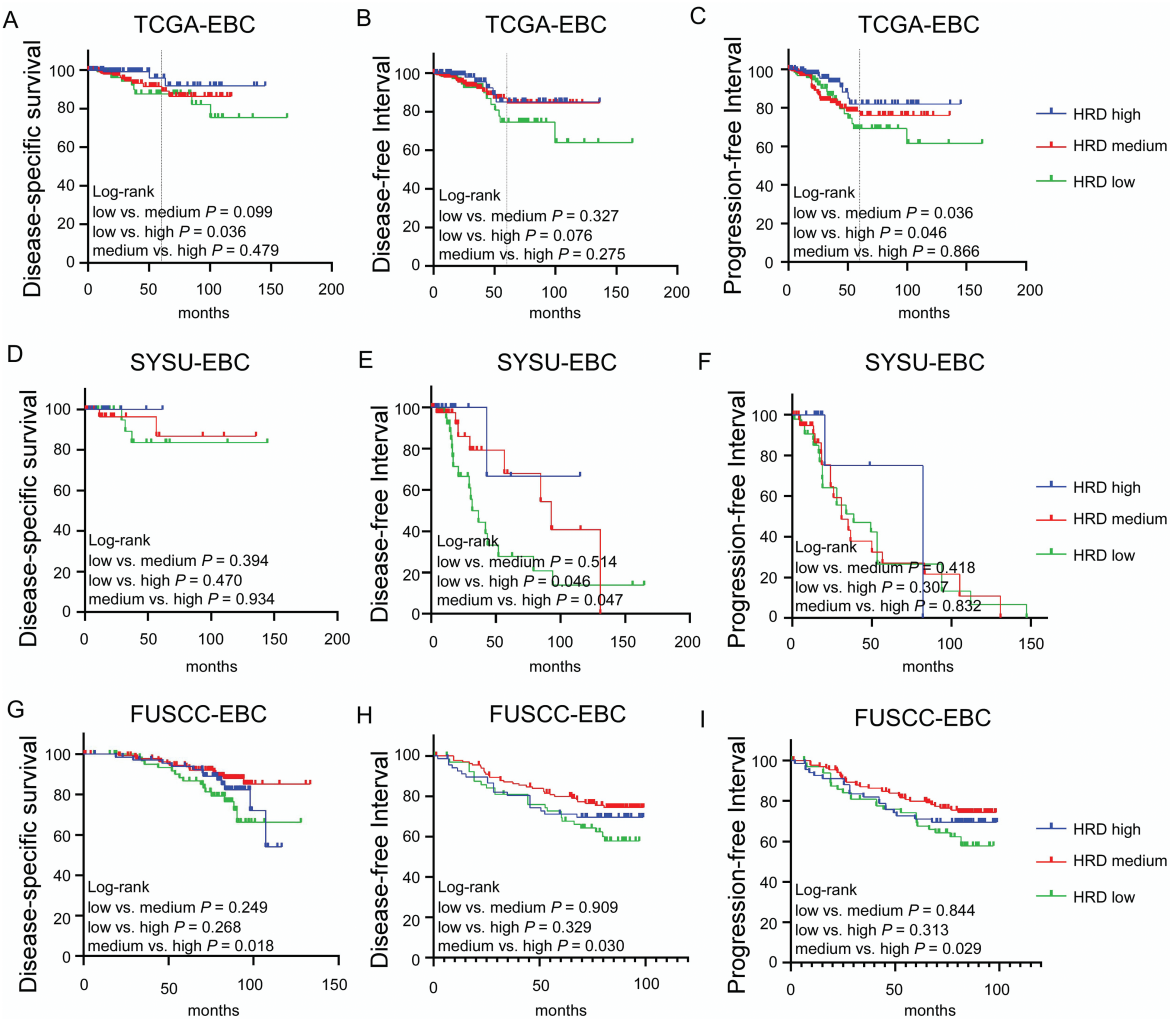
(**A**-**I**) Kaplan-Meier curve analysis of patients with HER2-EBC with different HRD status and long-term survival in different cohorts.

Figure 3 shows the Kaplan-Meier curves for DSS, DFI, and PFI for HER2-low EBC. Overall, there was a statistically significant difference in DSS (*P* = .036) and PFI (*P* = .046) between patients with HRD-low and HRD-high, and a high HRD score was marginally and significantly associated with poor DFI in patients with HER2-low EBC (Figure 3A-3C). Indeed, patients with HRD-high EBC showed relatively worse outcomes, with 5-year DSS and PFI rates of approximately 88% and 69%, respectively. In contrast, HRD-low was associated with a better prognosis, with 5-year DSS and PFI rates of approximately 96% and 82%, respectively. In addition, HRD-medium and HRD-high patients had worse PFIs than HRD-low patients (*P *= .036). Regarding DFI, no significant differences (*P* = .327 and *P *= .275, respectively) were observed in different HRD score subgroups (Fig. 3B). In addition, we analyzed the relationship between different HRD status and prognosis in SYSU-EBC and FUSCC-EBC patients, respectively. In SYSU-EBC cohort, the patients with HER2-low with HRD-low and HRD-medium had significantly better DFI (*P* = .046; *P* = .047) than those with HRD-high, but HRD states did not associate with the DSS and PFI in SYSU cohort (Fig. 3D-3F). Consistently, the same results were also observed in the FUSCC cohort. The OS (*P* = .018), DFI (*P* = .030), and PFI (*P* = .029) of patients with HRD-medium were significantly better than those with HRD-high (Fig. 3G-3I). To obtain a deeper understanding of HER2-low breast cancers, we took HR status and lymph nodes status into consideration in the subsequent analysis. In the HR-positive subgroup, patients with HRD-low had marginally significantly better DFI (*P *= .052) than patients with HRD-high EBC, and better PFI (*P *= .021) than patients with HRD-medium (Supplementary Fig. S4A-S4C). In the N0-N1 subgroup, patients with HRD-low had marginally significantly better DFI than patients with HRD-medium and HRD-high EBC, and patients with HRD-low also had better PFI than patients with HRD-medium (Supplementary Fig. S4D-F). In conclusion, HRD-low subgroup of patients with HER2-low EBC has a better survival prognosis in the TCGA, SYSU, and FUSCC cohorts, especially in patients with HR positive or fewer lymph node metastases. In addition, we also explored the relationship between HRD status and prognosis in patients with HER2-0 EBC. The results showed that there was no relation between HRD status and survival in patients with HER2-0 EBC (Supplementary Fig. S5). These indicate that patients with HER2-low EBC are a special subgroup, and HRD status is a potential good prognostic marker for patients with HER2-low EBC.

### Factors Affecting Prognosis in Patients With HER2-Low EBC

Next, we used univariate Cox regression to explore factors affecting DSS and DFI. Patients with positive HR status (DSS: HR = 0.42, 95% CI: 0.19-0.94, *P* *=* .035; DFI: HR = 0.41, 95% CI: 0.21-0.82, *P = *.012) were more favorable for obtaining DSS and DFI than those patients with negative HR status. Patients with cN2-N3 (DSS, HR = 2.63, 95% CI: 1.17-5.91, *P = *.019; DFI: HR = 3.57, 95% CI: 1.81-7.02, *P < *.001) or HRD-high status (HR = 3.74, 95% CI: 1.03-13.60, *P = *.045) were susceptible to DSS or DFI (Table 2). In addition, other clinicopathological features of patients with HER2-low EBC were not associated with DSS or DFI. We found that cN2-N3 was significantly associated with DSS after correction for HR and HRD status and was an independent factor for DSS (HR = 2.91, 95% CI: 1.25-6.78, *P *= .013, Table 2). Patients with cN2-N3 (HR = 0.33, 95% CI: 0.17-0.67, *P *= .002) and positive HR status (HR = 4.23, 95% CI: 2.12-8.45, *P* < .001) were also significantly associated with DFI after correction (Table 2). As shown in Table 2, patients with positive HR status were more favorable for obtaining a good PFI than those with negative status (HR = 0.55, 95% CI: 0.32-0.95, *P = *.033). However, age ≥ 60 (HR = 1.89, 95% CI: 1.14-3.15, *P *= .014), cN2-N3 (HR = 2.21, 95% CI: 1.29-3.80, *P *= .004), HRD-medium (HR = 2.11, 95% CI: 1.04-4.30, *P* = .040), and HRD-high (HR = 2.21, 95% CI: 1.03-4.76, *P *= .042) were important factors in obtaining a poor PFI. After including multivariate Cox regression analysis, we found that age ≥ 60 (HR = 2.41, 95% CI: 1.43-4.06, *P *= .001), cN2-N3 (HR = 2.55, 95% CI: 1.46-4.44, *P *= .001), HR (HR = 0.46, 95% CI: 0.24-0.87, *P *= .016), or HRD-medium (HR = 2.15, 95% CI: 1.04-4.41, *P *= .038) remained meaningful for PFI after correction for HRD-high. We also found similar results for cN2-N3 status (DSS, HR = 4.14, 95% CI: 1.59-10.81, *P* = .004; DFI HR = 4.22, 95% CI: 1.91-9.33, *P* < .001; PFI, HR = 2.44, 95% CI: 1.11-4.93, *P* = .013) and hormone receptor positives (DSS, HR = 0.23, 95% CI: 0.10-0.53, *P* = .001; DFI, HR = 0.33, 95% CI: 0.16-0.66, *P* = .013) in the HER2-0 subgroup, which remained significant after multivariate adjustment (Supplementary Table S3).

**Table 2.** Univariate and multivariate analysis of the risk factors for DSS, DFI, and PFI among patients with HER2-low TCGA-EBC.

Variables	DSS				DFI				PFI			
	Univariable analysis		Multivariable analysis		Univariable analysis		Multivariable analysis		Univariable analysis		Multivariable analysis	
	HR (95% CI)	*P*-value	HR (95% CI)	*P*-value	HR (95% CI)	*P*-value	HR (95% CI)	*P*-value	HR (95% CI)	*P*-value	HR (95% CI)	*P*-value
Age: ≥60 vs <60	1.20 (0.61-2.33)	0.598	—	—	1.67 (0.77-3.63)	0.194	—	—	1.89 (1.14-3.15)	0.014	2.41 (1.43-4.06)	0.001
Tumor size: T3-T4 vs T1-T2	2.23 (0.96-5.14)	0.061	—	—	1.945 (0.87-4.34)	0.104	—	—	1.62 (0.90-2.92)	0.106	—	—
Lymph nodes: N2-N3 vs N0-N1	2.63 (1.17-5.91)	0.019	2.91 (1.25-6.78)	0.013	3.57 (1.81-7.02)	<0.001	4.23 (2.12-8.45)	<0.001	2.21 (1.29-3.80)	0.004	2.55 (1.46-4.44)	0.001
IHC: 2 + vs 1+	0.54 (0.25-1.18)	0.124	—	—	0.66 (0.34-1.29)	0.225	—	—	0.74 (0.45-1.23)	0.241	—	—
HR status: positive vs negative	0.42 (0.19-0.94)	0.035	0.39 (0.15-1.01)	0.052	0.41 (0.21-0.82)	0.012	0.33 (0.17-0.67)	0.002	0.55 (0.32-0.95)	0.033	0.46 (0.24-0.87)	0.016
HRD status: medium vs low	2.80 (0.80-9.85)	0.108	/	/	1.62 (0.63-4.14)	0.317	/	/	2.11 (1.04-4.30)	0.040	2.15 (1.04-4.41)	0.038
HRD status: high vs low	3.74 (1.03-13.60)	0.045	2.04 (0.51-8.20)	0.315	2.41 (0.92-6.36)	0.075	—	—	2.21 (1.03-4.76)	0.042	2.71 (0.73-4.03)	0.217
HRR mutations status: YES vs NO	1.42 (0.46-4.41)	0.543	—	—	1.07 (0.40-2.83)	0.895	—	—	0.72 (0.30-1.70)	0.451	—	—
BRCA1/2 mutations status: YES vs NO	1.58 (0.21-12.00)	0.657	—	—	1.49 (0.69-3.20)	0.215	—	—	1.38 (0.33-5.71)	0.657	—	—

Abbreviations: DSS, disease-specific survival; DFI, disease-free interval; PFI, progression-free interval; TCGA, The Cancer Genome Atlas dataset; HRD, homologous recombination defect score; HER2, human epidermal growth factor receptor 2; IHC, immunohistochemistry; HR, hormone receptor; HRRGs, homologous recombination repair genes; BRCA, breast cancer susceptibility gene.

### Subgroup Analysis of Patients With HER-Low EBC

**Figure 4.**
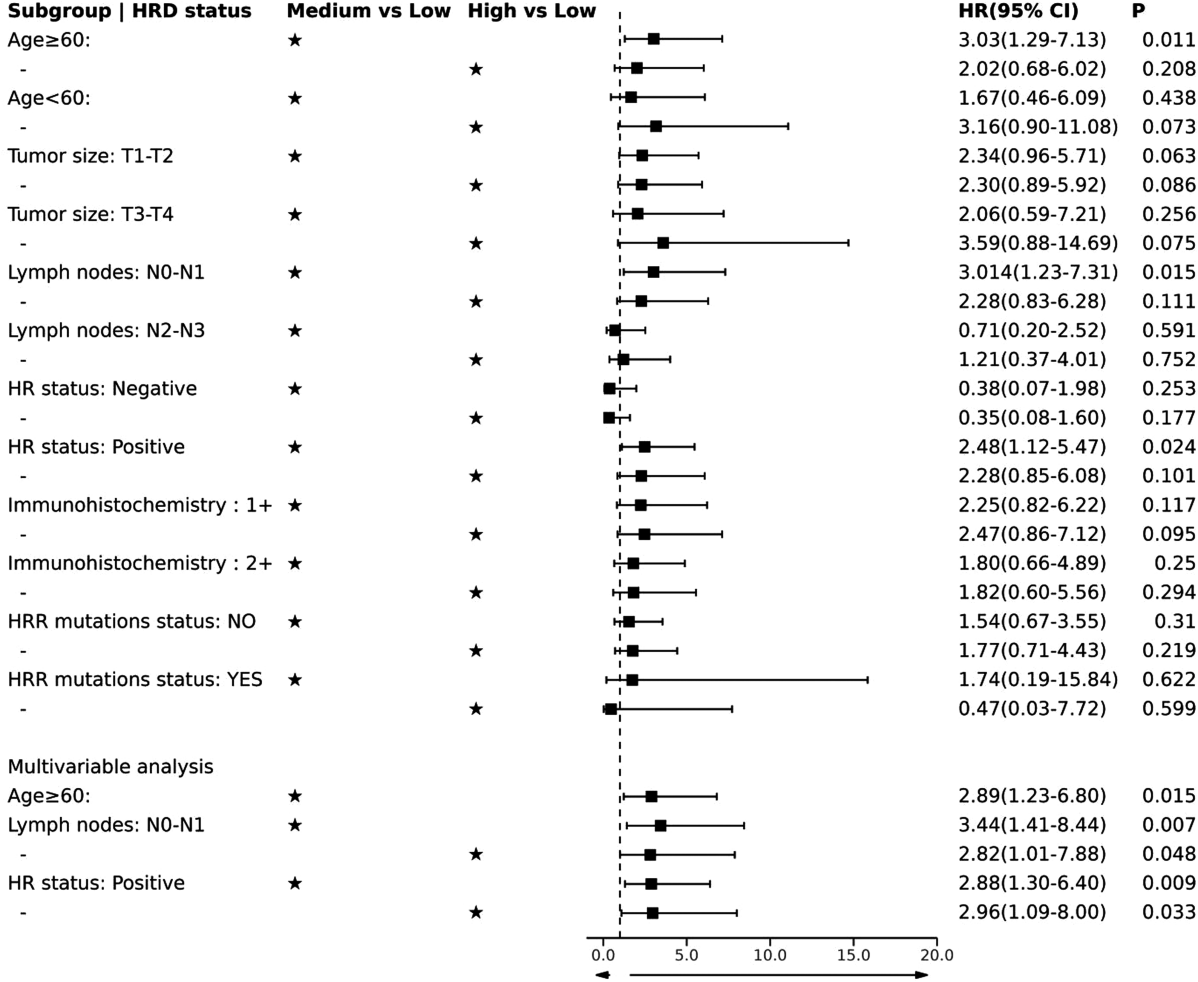
Results of HRD status in subgroup analysis at PFI in HER2-low TCGA-EBC.

Since our analysis indicated that age, T stage, LN stage, and HR status are independent risk factors for DSS, DFI, and PFI in patients with HER2-low EBC, we performed subgroup analysis to further explore their association with HRD scores. We stratified subgroup analysis of patients with HER-low EBC by age, T status, LN status, and HR status to explore whether HRD score status shows unique significance in a specific population. We found that HRD-high and HRD-medium remained statistically significant for PFI in patients with cN0-N1 (HRD-high, HR = 2.82, 95% CI: 1.01-7.88, *P *= .048; HRD-medium, HR = 3.44, 95% CI: 1.41-8.44, *P *= .007) and positive HR status (HRD-high, HR = 2.96, 95% CI: 1.09-8.00, *P *= .033; HRD-medium, HR = 2.88, 95% CI: 1.30-6.40, *P *= .009) after correction by multivariate Cox regression analysis (Fig. 4; Supplementary Tables S5-S6). HRD-medium (HR = 2.89, 95% CI: 1.23-6.80, *P *= .015, Fig. 4; Supplementary Table S3) remained statistically significant for PFI after correction in the age ≥ 60 HER2-low EBC subgroup. Both HRD-high and HRD-medium were present in the 3 subgroups of interest as adverse prognostic factors affecting PFI (Fig. 4). In addition, HRD status in other clinicopathological feature subgroups of the patients was not found to affect DSS or DFI (Supplementary Figs. S6-S7 and Table S4-S7).

## Discussion

More than 50% of EBC tumors are in fact the HER2-low subtype, which has a low NAC PCR rate and poor survival. The importance of HRD in predicting and treating tumors has been gradually recognized because of its role in heritable genomic changes and the development of cancer.^39^ This is the first study to evaluate the relationship between HRD levels and the prognosis of patients with HER2-low EBC. In this retrospective and observational study, we found that HRD scores ranging from 8 to 33 or over 33 are significant risk factors for PFI, and higher HRD scores are associated with poor long-term outcomes.

Several studies have investigated the prognostic role of the HRD score in other subtypes of EBC.^19,20,40-42^ Sharma et al^21^ investigated the prognostic role of HRD status in patients with triple-negative breast cancer (TNBC) from the SWOG S9313 trial, and the results indicated that a high HRD score was associated with shorter DFS (*P *= .049), while a high HRD score marginally predicted poor OS (*P = *.073). The Geparsix trial also indicated that the HRD score was a predictor of treatment response in non-tumor tissue *BRCA* mutation-detected patients with TNBC.^19,20^ Consistently, our study found that patients with a medium HRD score had significantly worse DSS, and patients with medium HRD or high HRD scores had a significantly worse PFI. Patients with high HRD scores had the worst PFI, partly due to the association between the HRD score and genomic instability, which is crucial for EBC progression and closely related to the activation of homologous recombination pathways.^41,42^

Furthermore, we also found that HRD-high and HRD-medium significantly affected PFI as poor prognostic factors compared with HRD-low in the HR-positive subgroup. In our analysis, HRD expression was higher in HR + subtypes, as expected, with significant differences between HER2-low/HR + and HER2-low/HR−. This may be explained in the following 2 ways. First, studies have shown that approximately 70% of patients with breast cancer are hormone receptor positive (HR+), and most of them are of the HER2−/HR + subtype.^43,44^ Second, patients with triple-negative breast cancer (TNBC) with pathogenic BRCA1/2 mutations had higher PCR rates with or without carboplatin in the GeparSixto study (66.7% vs 36.4%, OR 3.50; 95% CI: 1.39-8.84; *P* = .008).^19^ Our results appear to complement the study, where HR+/HRD-high predicted a worse prognostic outcome. The latest analysis of the breast cancer genome by the Harvard University research team shows that estrogen processing can directly induce DNA double-strand breaks in the region where the estrogen receptor is located so that cells repair the break through intrachromosomal rearrangement and directly induce the copy number amplification of oncogenes involved in cancer development and development.^45,46^ Therefore, patients with hormone receptor-positive breast cancer may be more likely to obtain high DNA damage and high HRD scores. Moreover, studies have shown that 3% of untreated patients and 30% of patients after endocrine therapy may develop primary and secondary resistance.^47,48^Taken together, our findings reinforce current speculation about hormone receptors and DNA repair, deserve deeper exploration, and may be particularly useful for future therapeutic developments.

When compared with HER2-0 tumors breast cancer, HER2-low breast cancer is a newly defined subtype of breast cancer harboring higher *ERBB2* alleles and less *ERBB2* hemi deletions, but there is no significant difference on genomic alterations or tumor mutation burden between HER2-low and HER2-0 breast cancer after multiple hypothesis testing.^49^ Interestingly, we found HRD status associates with prognosis of patients with HER2-low EBC, while no significant difference was found in the prognosis of HER-0 tumors in our study. This could partially be explained by the more enrollment of HR-negative patients in HER2-0 group and enrollment of stage IV patients in both groups, as genetic alteration patterns are significantly different in HR + breast cancer compared with HR− breast cancer^50^ and genetic alteration patterns are significantly different in EBC compared with advanced breast cancer.^51^ This implies that HER2-low EBC may exhibit a distinct genetic alteration from HER2-0 EBC, but this needs to be confirmed by large-scale genome sequencing. Nevertheless, the HRD score is a good predictor of survival outcomes in patients with HER2-lowEBC, which may lay a good foundation for guiding personalized treatment of patients with HER2-low EBC. In conclusion, patients with HER-low EBC with high HRD or medium HRD scores have a shorter PFI than patients with low HRD scores, especially HR+/HER2-low EBC patients.

Homologous recombination defects play a pivotal role in tumorigenesis by causing compromised repair of double-stranded DNA breaks. While they may potentially trigger and enhance various tumor immune responses, a comprehensive comprehension of the tumor microenvironment linked to HRD remains currently uncertain. Therefore, we implemented the comparison of the expression level of TIICs among different HRD status of HER2-low EBC patients to attain the potential implications for immunotherapy. The results unveiled that a wide range of immune cell activation signatures is enriched in HRD-high cases of HER2-low EBC, which indicated that patients with BC with higher HRD might obtain a more enhanced response to therapies targeting these checkpoints. Otherwise, the cytotoxicity caused by the escape of tumor cells when the immune system is destroyed can be mitigated by continued activation of these immune cells.^52^ There has been evidence that the abnormal homologous recombination repair pathway of DNA double-strand breaks is closely related to the tumor immune microenvironment.^53^ Consequently, the landscape of TME among HRD groups signified that CD8 T+ cell, plasma B cell and macrophages M1 were notably strengthened for patients with HER2-low EBC in the HRD-high, while activated mast cells, monocyte, cancer associated fibroblast and hematopoietic stem cell were markedly activated in the HRD-low group. The immune-response-associated genomic features, including immune score and stroma score, correlated with HRD scores. In the preceding study, notable infiltration of T lymphocytes, including CD8+ T cells, was observed in tumor tissues attributed to homologous recombination repair defects,^54,55^ aligning with our own research findings. Concerning the impact of macrophages on tumors, they are commonly recognized for their anti-tumor effects.^56^ Emerging evidence suggests that HRD can influence the inflammatory milieu within tumors, shaping the polarization status of macrophages and intricately linking to heightened activity of M1 macrophages.^57,58^ The heightened expression of mast cells, monocytes, cancer-associated fibroblasts, and hematopoietic stem cells in the HRD-low lacks a comprehensive explanation in current studies. We guess that HRD-low expression may signify more effective immune regulation, reduced inflammation levels, and a relatively stable tumor microenvironment, potentially enhancing the functionality of cancer-related cells (such as fibroblasts) and immune cells (such as mast cells and monocytes), enabling active engagement in immune responses. Collectively, these findings imply distinct immune landscapes across various HRD states in patients with HER2-low EBC, potentially unveiling novel implications for immunotherapeutic interventions.

There are still a number of limitations in this study: (i) this is a retrospective study that potentially has biases; (ii) only somatic gene mutations were included in this study. Somatic mutation data only partially explain the effect of the defective state of homologous recombination. (iii) Since the data in this study were mainly from the TCGA public database, the included population was mostly Caucasian, which may not be a good explanation for the possible racial heterogeneity. (iv) More patient data and sufficient follow-up time are needed to strengthen the results of this study. (v) Lack of correction for prognostic outcomes with treatment options.

## Supplementary Material

oyae021_suppl_Supplementary_Figure_S1

oyae021_suppl_Supplementary_Figure_S2

oyae021_suppl_Supplementary_Figure_S3

oyae021_suppl_Supplementary_Figure_S4

oyae021_suppl_Supplementary_Figure_S5

oyae021_suppl_Supplementary_Figure_S6

oyae021_suppl_Supplementary_Figure_S7

oyae021_suppl_Supplementary_Table_S1

oyae021_suppl_Supplementary_Table_S2

oyae021_suppl_Supplementary_Table_S3

oyae021_suppl_Supplementary_Table_S4

oyae021_suppl_Supplementary_Table_S5

oyae021_suppl_Supplementary_Table_S6

oyae021_suppl_Supplementary_Table_S7

oyae021_suppl_Supplementary_Figure_Captions

## Data Availability

The datasets analyzed during the current study are available from the corresponding author on reasonable request. The dataset supporting the conclusions of this article is available in the Cancer Genome Atlas (TCGA) database (https://portal.gdc.cancer.gov), UCSC Xena (https://xenabrowser.net), and cBioPortal (http://www.cbioportal.org).
