# Supplementary figures and images for: Association between Homologous Recombination Repair Defect Status and Long-Term Prognosis of Early HER2-Low Breast Cancer: A Retrospective Cohort Study
Source: Oncologist. 2024 Feb 16;29(7):e864–76. doi: 10.1093/oncolo/oyae021 (PMC11224982; doi:10.1093/oncolo/oyae021)

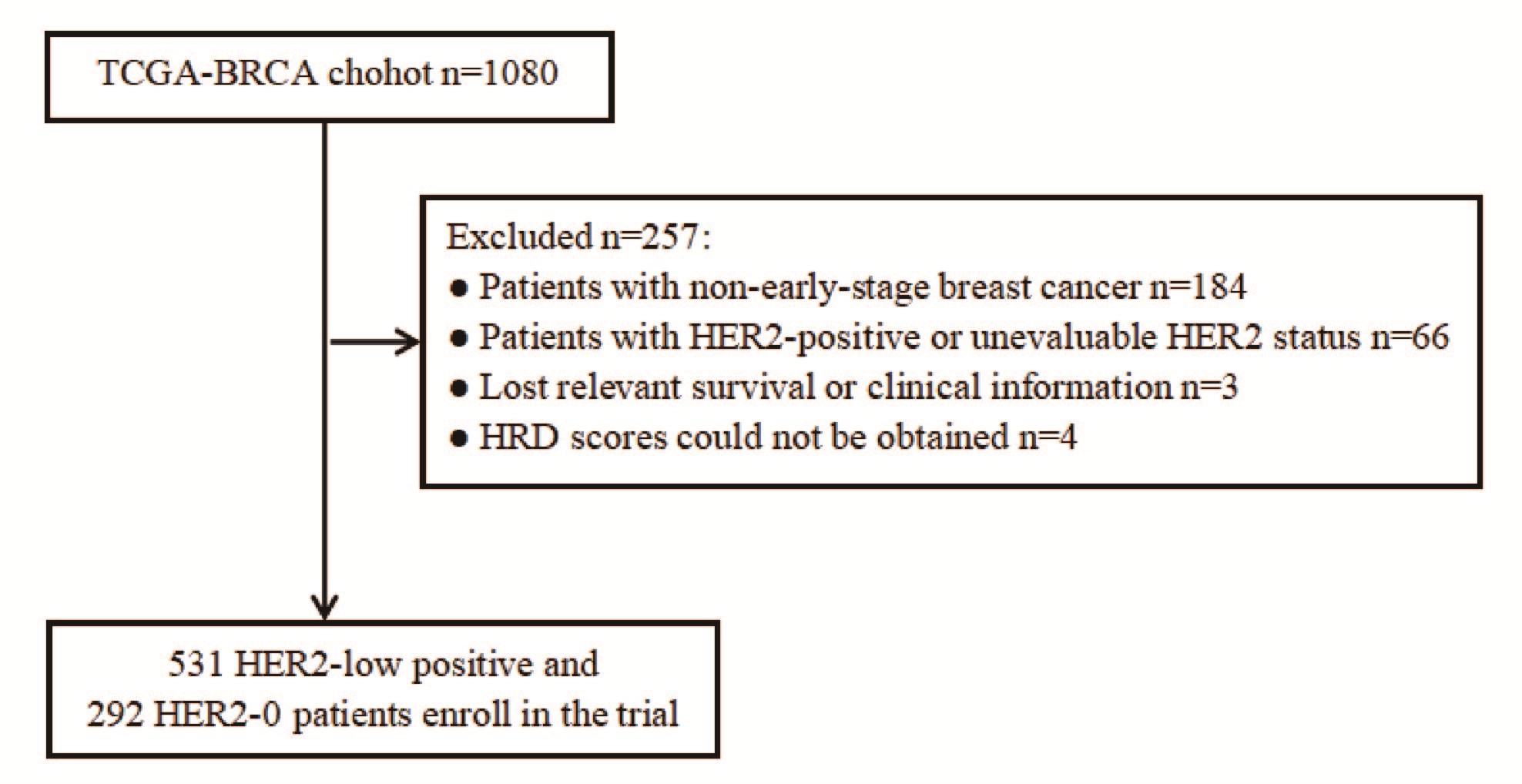

Supplement: oyae021_suppl_Supplementary_Figure_S1 [file oyae021_suppl_supplementary_figure_s1.jpeg]

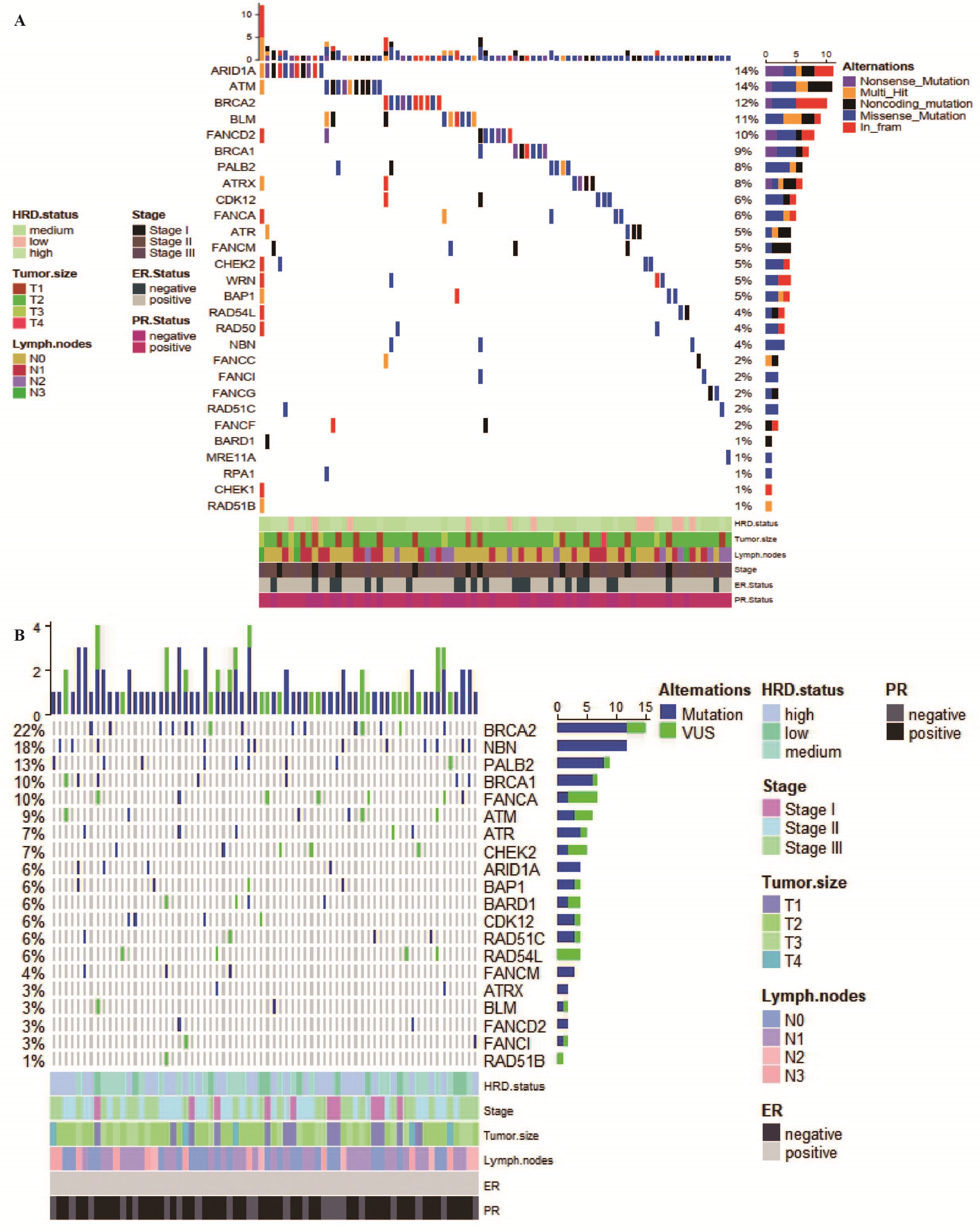

Supplement: oyae021_suppl_Supplementary_Figure_S2 [file oyae021_suppl_supplementary_figure_s2.jpeg]

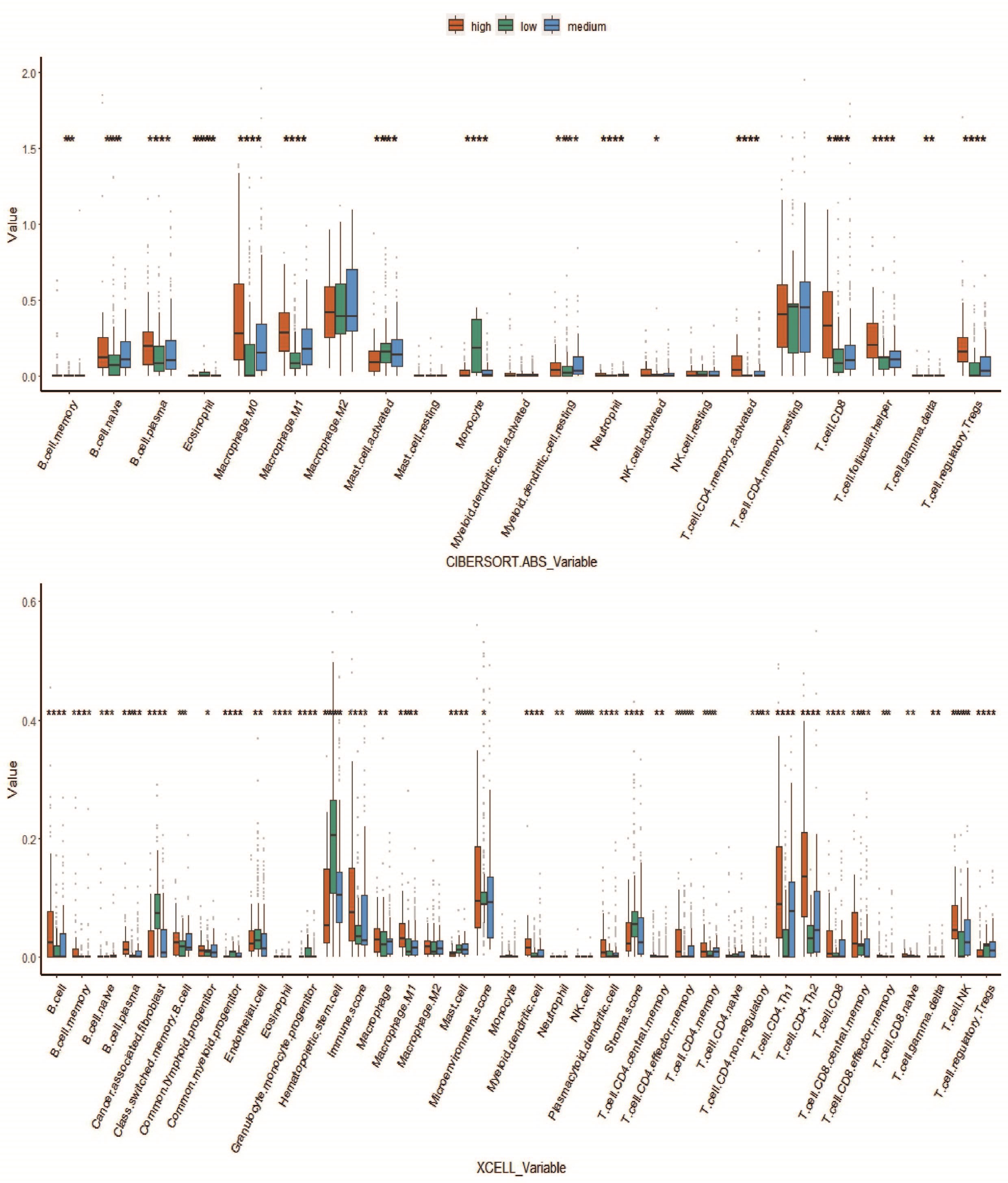

Supplement: oyae021_suppl_Supplementary_Figure_S3 [file oyae021_suppl_supplementary_figure_s3.jpeg]

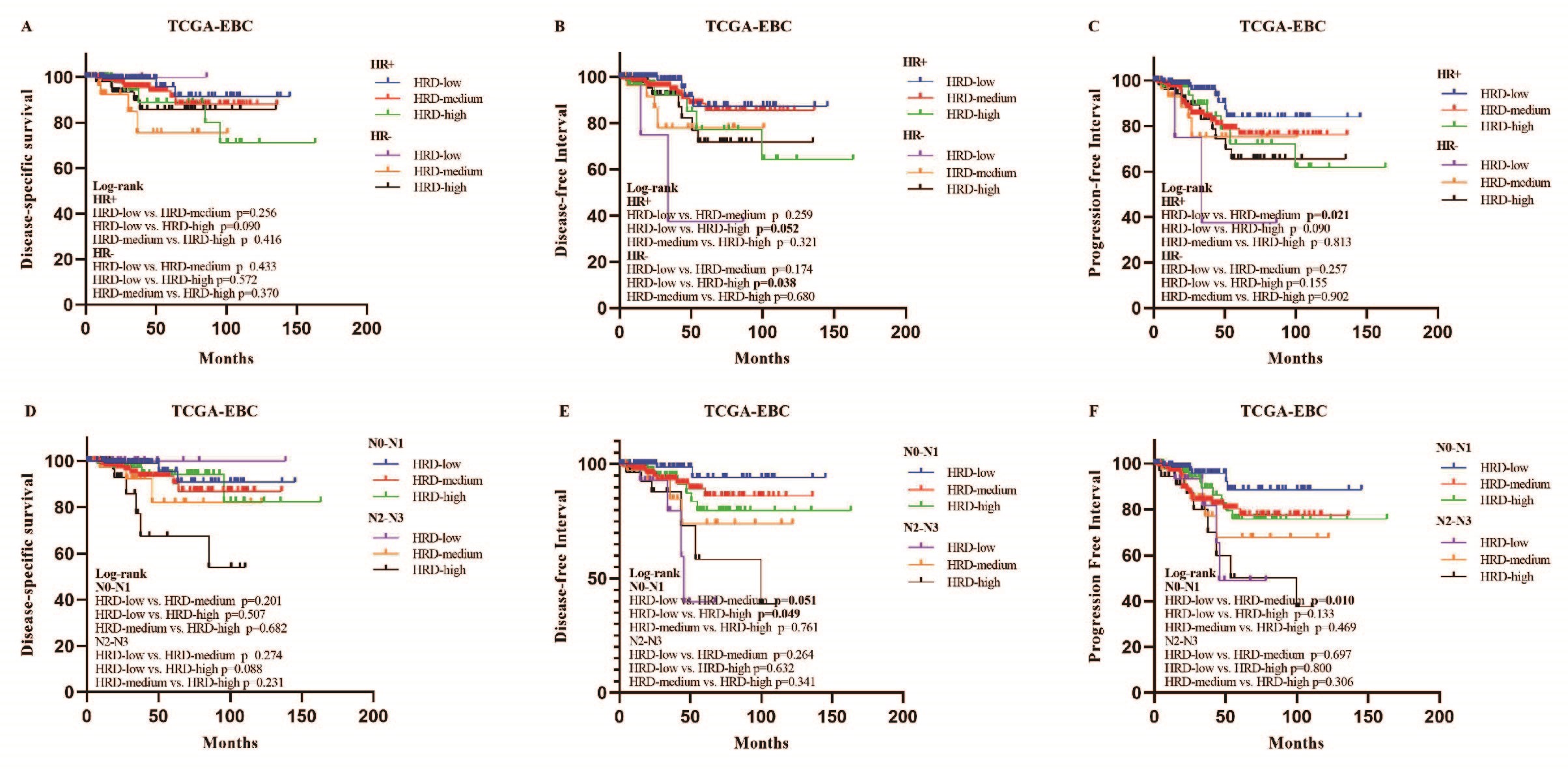

Supplement: oyae021_suppl_Supplementary_Figure_S4 [file oyae021_suppl_supplementary_figure_s4.jpeg]

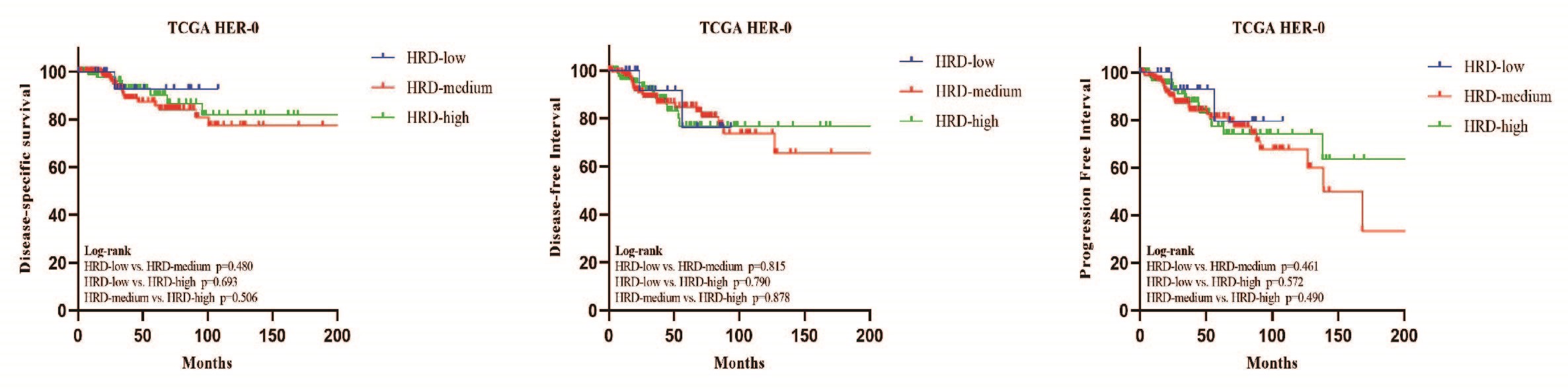

Supplement: oyae021_suppl_Supplementary_Figure_S5 [file oyae021_suppl_supplementary_figure_s5.jpeg]

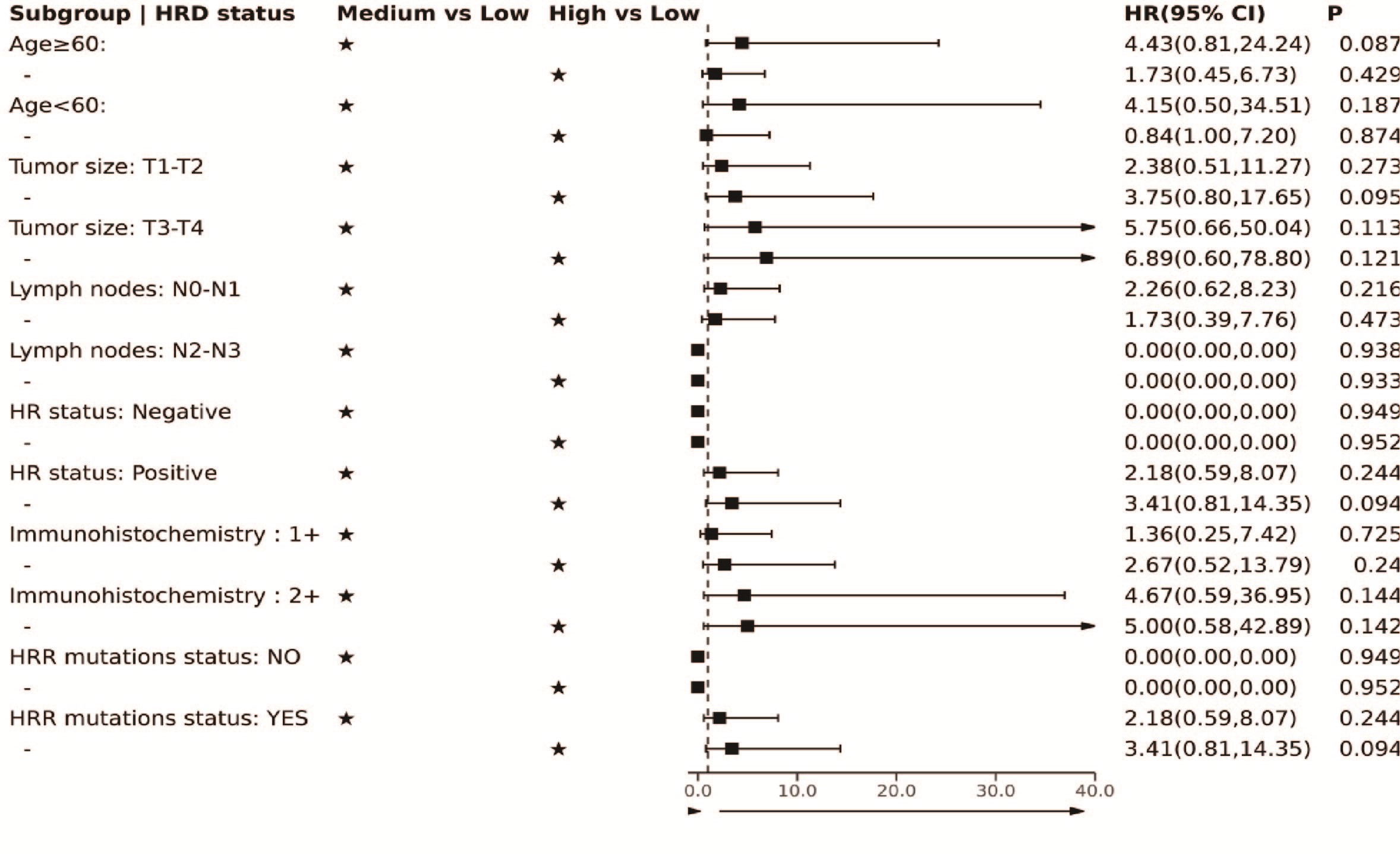

Supplement: oyae021_suppl_Supplementary_Figure_S6 [file oyae021_suppl_supplementary_figure_s6.jpeg]

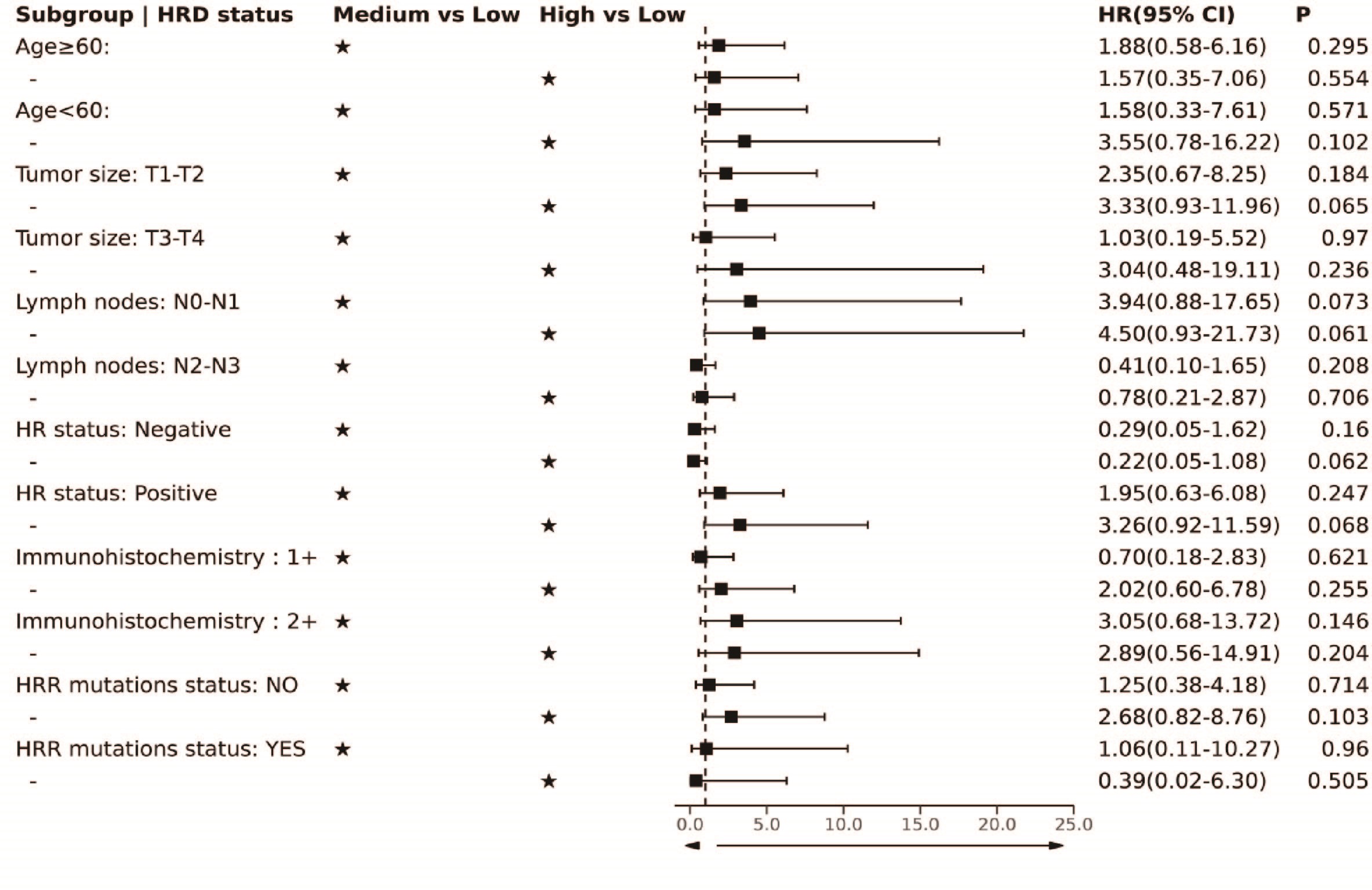

Supplement: oyae021_suppl_Supplementary_Figure_S7 [file oyae021_suppl_supplementary_figure_s7.jpeg]
