# Supplementary material for: Association between Homologous Recombination Repair Defect Status and Long-Term Prognosis of Early HER2-Low Breast Cancer: A Retrospective Cohort Study
Source: Oncologist. 2024 Feb 16;29(7):e864–76. doi: 10.1093/oncolo/oyae021 (PMC11224982; doi:10.1093/oncolo/oyae021)
Supplement: oyae021_suppl_Supplementary_Table_S1 [file oyae021_suppl_supplementary_table_s1.docx]

**Supplementary Table1: Clinical baseline characteristics of different HRD states in SYSU-EBC and FUSSC-EBC HER2-low cohorts.**

| Clinical Characteristic | SYSU HER2-low | | | | |  | FUSSC HER2-low | | | | |
| --- | --- | --- | --- | --- | --- | --- | --- | --- | --- | --- | --- |
|  | All Patients (n=110) | Number of patients (%) | | |  |  | All Patients (n=270) | Number of patients (%) | | |  |
|  |  | HRD ≤ 8 (n=17) | HRD 9-33 (n=45) | HRD>33 (n=48) | *P* value |  |  | HRD ≤ 8 (n=68) | HRD 9-33 (n=135) | HRD>33 (n=67) | *P* value |
| Age (years) |  |  |  |  | 0.263 |  |  |  |  |  | 0.897 |
| < 60 | 97(88.18%) | 16(94.12%) | 37(82.22%) | 44(91.67%) |  |  | 201(74.44%) | 52(76.47%) | 100(74.07%) | 49(73.13%) |  |
| ≥ 60 | 13(11.82%) | 1(5.88%) | 8(17.78%) | 4(8.33%) |  |  | 69(25.56%) | 16(23.53%) | 35(25.93%) | 18(26.87%) |  |
| TNM stage |  |  |  |  | 0.085 |  |  |  |  |  | 0.171 |
| Stage I-II | 72(65.45%) | 12(70.59%) | 34(75.56%) | 26(54.17%) |  |  | 181(67.04%) | 45(66.18%) | 85(62.96%) | 51(76.12%) |  |
| Stage III | 38(34.55%) | 5(29.41%) | 11(24.44%) | 22(45.83%) |  |  | 89(32.96%) | 23(33.82%) | 50(37.04%) | 16(23.88%) |  |
| Tumor size |  |  |  |  | 0.800 |  |  |  |  |  | 0.225 |
| T1-T2 | 78(70.91%) | 11(64.71%) | 33(73.33%) | 34(70.83%) |  |  | 269(99.63%) | 67(98.53%) | 135(100.00%) | 67(100.00%) |  |
| T3-T4 | 32(29.09%) | 6(35.29%) | 12(26.67%) | 14(29.17%) |  |  | 1(0.37%) | 1(1.47%) | 0(0.00%) | 0(0.00%) |  |
| Lymph nodes |  |  |  |  | 0.036 |  |  |  |  |  | 0.493 |
| N0-N1 | 86(78.18%) | 15(88.24%) | 39(86.67%) | 32(66.67%) |  |  | 192(71.11%) | 45(66.18%) | 100(74.07%) | 47(70.15%) |  |
| N2-N3 | 24(21.82%) | 2(11.76%) | 6(13.33%) | 16(33.33%) |  |  | 78(28.89%) | 23(33.82%) | 35(25.93%) | 20(29.85%) |  |
| IHC |  |  |  |  | 0.732 |  |  |  |  |  | 0.016 |
| 1+ | 46(41.82%) | 7(41.18%) | 17(37.78%) | 22(45.83%) |  |  | 161(59.63%) | 34(50.00%) | 92(68.15%) | 35(52.24%) |  |
| 2+ | 64(58.18%) | 10(58.82%) | 28(62.22%) | 26(54.17%) |  |  | 109(40.37%) | 34(50.00%) | 43(31.85%) | 32(47.76%) |  |
| HR status |  |  |  |  | 0.738 |  |  |  |  |  | / |
| Negative | 8(7.27%) | 2(11.76%) | 3(6.67%) | 3(6.25%) |  |  | 0(0.00%) | 0(0.00%) | 0(0.00%) | 0(0.00%) |  |
| Positive | 102(92.73%) | 15(88.24%) | 42(93.33%) | 45(93.75%) |  |  | 270(100.00%) | 68(100.00%) | 135(100.00%) | 67(100.00%) |  |
| HRRGs  mutations status |  |  |  |  | 0.515 |  |  |  |  |  | / |
| NO | 41(37.27%) | 7(41.18%) | 19(42.22%) | 15(31.25%) |  |  | / | / | / | / |  |
| YES | 69(62.73%) | 10(58.82%) | 26(57.78%) | 33(68.75%) |  |  | / | / | / | / |  |
| BRCA1/2  mutation status |  |  |  |  | 0.256 |  |  |  |  |  | / |
| NO | 86(78.18%) | 14(82.35%) | 38(84.44%) | 34(70.83%) |  |  | / | / | / | / |  |
| YES | 24(21.82%) | 3(17.65%) | 7(15.56%) | 14(29.17%) |  |  | / | / | / | / |  |

Abbreviation: SYSU, Sun Yat-sen memorial hospital; FUSCC, Fudan University Shanghai Cancer Center; EBC, early breast cancer; HRD, homologous recombination defect score; IHC, Immunohistochemistry; HR, hormone receptor; HRRGs, Homologous Recombination Repair Genes; BRCA, breast cancer susceptibility gene.
