# Supplementary material for: Association between Homologous Recombination Repair Defect Status and Long-Term Prognosis of Early HER2-Low Breast Cancer: A Retrospective Cohort Study
Source: Oncologist. 2024 Feb 16;29(7):e864–76. doi: 10.1093/oncolo/oyae021 (PMC11224982; doi:10.1093/oncolo/oyae021)
Supplement: oyae021_suppl_Supplementary_Table_S2 [file oyae021_suppl_supplementary_table_s2.docx]

**Supplementary Table 2: Clinical baseline characteristics among different status of the HRD in HER2-0 TCGA-EBC.**

| TCGA HER2-0  Clinical Characteristic | All Patients (n=292) | Number of patients (%) | | |  |
| --- | --- | --- | --- | --- | --- |
|  |  | HRD ≤ 8 (n=19) | HRD 9-33 (n=169) | HRD>33 (n=104) | *P* value |
| Age (years) |  |  |  |  | 0.520 |
| < 60 | 170(58.22%) | 10(52.63%) | 95(56.21%) | 65(62.50%) |  |
| ≥ 60 | 122(41.78%) | 9(47.37%) | 74(43.79%) | 39(37.50%) |  |
| TNM stage |  |  |  |  | 0.581 |
| Stage I-II | 225(77.05%) | 16(84.21%) | 127(75.15%) | 82(78.85%) |  |
| Stage III | 67(22.95%) | 3(15.79%) | 42(24.85%) | 22(21.15%) |  |
| Tumor size |  |  |  |  | 0.838 |
| T1-T2 | 238(81.51%) | 16(84.21%) | 139(82.25%) | 83(79.81%) |  |
| T3-T4 | 54(18.49%) | 3(15.79%) | 30(7.75%) | 21(20.19%) |  |
| Lymph nodes |  |  |  |  | 0.147 |
| N0-N1 | 248(84.93%) | 16(84.21%) | 138(81.66%) | 94(90.38%) |  |
| N2-N3 | 44(15.07%) | 3(15.79%) | 31(18.34%) | 10(9.62%) |  |
| HR status |  |  |  |  | 0.521 |
| Negative | 111(38.01%) | 5(26.32%) | 67(39.64%) | 39(37.50%) |  |
| Positive | 181(61.99%) | 14(73.68%) | 102(60.36%) | 65(62.50%) |  |
| HRRGs mutations status  (include BRCA1/2) |  |  |  |  | 0.011 |
| NO | 286(97.95%) | 17(89.47%) | 165(97.63%) | 104(100.00%) |  |
| YES | 6(2.05%) | 2(10.53%) | 4(2.37%) | 0(0.00%) |  |

Abbreviation: TCGA, The Cancer Genome Atlas dataset; EBC, early breast cancer; HRD, homologous recombination defect score; IHC, Immunohistochemistry; HR, hormone receptor; HRRGs, Homologous Recombination Repair Genes; BRCA, breast cancer susceptibility gene.
