# Supplementary material for: Association between Homologous Recombination Repair Defect Status and Long-Term Prognosis of Early HER2-Low Breast Cancer: A Retrospective Cohort Study
Source: Oncologist. 2024 Feb 16;29(7):e864–76. doi: 10.1093/oncolo/oyae021 (PMC11224982; doi:10.1093/oncolo/oyae021)
Supplement: oyae021_suppl_Supplementary_Table_S3 [file oyae021_suppl_supplementary_table_s3.docx]

**Supplementary Table 3: Univariate and Multivariate analysis of the risk factors for DSS, DFI and PFI among HER2-0 TCGA-EBC patients**

| TCGA HER2-0  Variables | DSS | | | | DFI | | | | PFI | | | |
| --- | --- | --- | --- | --- | --- | --- | --- | --- | --- | --- | --- | --- |
|  | Univariable analysis | | Multivariable analysis | | Univariable analysis | | Multivariable analysis | | Univariable analysis | | Multivariable analysis | |
|  | HR (95% CI) | *P* value | HR (95% CI) | *P* value | HR (95% CI) | *P* value | HR (95% CI) | *P* value | HR (95% CI) | *P* value | HR (95% CI) | *P* value |
| Age: ≥60 *vs.* <60 | 1.22(0.56-2.66) | 0.615 | / | / | 1.07(0.54-2.11) | 0.843 | / | / | 1.09(0.60-1.95) | 0.784 | / | / |
| Tumor size:  T3-T4 *vs.* T1-T2 | 1.66(0.69-3.96) | 0.256 | / | / | 2.04(0.70-4.19) | 0.051 | / | / | 1.61(0.85-3.08) | 0.147 | / | / |
| Lymph nodes:  N2-N3 *vs.* N0-N1 | 2.67(1.07-6.66) | 0.035 | 4.14(1.59-10.81) | 0.004 | 3.13(1.46-6.71) | 0.003 | 4.22(1.91-9.33) | 0.000 | 2.44(1.21-4.93) | 0.013 | 2.44(1.21-4.93) | 0.013 |
| HR status:  Positive *vs.* Negative | 0.29(0.13-0.64) | 0.002 | 0.23(0.10-0.53) | 0.001 | 0.41(0.21-0.80) | 0.009 | 0.33(0.16-0.66) | 0.002 | 0.58(0.32-1.02) | 0.060 | / | / |
| HRD status:  medium *vs.* low | 2.02(0.27-15.21) | 0.494 | / | / | 1.23(0.29-5.24) | 0.783 | / | / | 1.76(0.42-7.39) | 0.438 | / | / |
| HRD status:  high *vs.* low | 1.52(0.19-12.16) | 0.694 | / | / | 1.17(0.26-5.23) | 0.841 | / | / | 1.42(0.32-6.25) | 0.642 | / | / |
| HRR mutations status  (INC BRCA1/2 mutation):  YES *vs.* NO | 3.06(0.72-12.97) | 0.129 | / | / | 3.57(0.85-14.92) | 0.082 | / | / | 2.05(0.85-8.50) | 0.321 | / | / |

Abbreviation: EBC, early breast cancer; TCGA, The Cancer Genome Atlas dataset; HRD, homologous recombination defect score; IHC, Immunohistochemistry; HR, hormone receptor; HRRGs, Homologous Recombination Repair Genes; BRCA, breast cancer susceptibility gene
