# Supplementary material for: Association between Homologous Recombination Repair Defect Status and Long-Term Prognosis of Early HER2-Low Breast Cancer: A Retrospective Cohort Study
Source: Oncologist. 2024 Feb 16;29(7):e864–76. doi: 10.1093/oncolo/oyae021 (PMC11224982; doi:10.1093/oncolo/oyae021)
Supplement: oyae021_suppl_Supplementary_Table_S4 [file oyae021_suppl_supplementary_table_s4.docx]

**Supplementary Table 4. Relationship of HRD and survival prognosis according to age subgroups in TCGA-EBC**

| **Variables** | **Age ≥60** | | | | **Age <60** | | | |
| --- | --- | --- | --- | --- | --- | --- | --- | --- |
|  | Univariable analysis | | Multivariable analysis | | Univariable analysis | | Multivariable analysis | |
|  | OR (95% CI) | *P* value | OR (95% CI) | *P* value | OR (95% CI) | *P* value | OR (95% CI) | *P* value |
|  | **DSS** | | | | | | | |
| Tumor size: T3-T4 *vs* T1-T2 | 1.94(0.60-5.88) | 0.243 | / | / | 2.22(0.60-8.20) | 0.233 | / | / |
| Lymph nodes: N2-N3 *vs* N0-N1 | 3.54(1.19-10.57) | 0.024 | 3.54(1.19-10.57) | 0.024 | 2.08(0.62-6.95) | 0.233 | / | / |
| HR status: Positive *vs* Negative | 0.33(0.10-1.05) | 0.061 | / | / | 0.42(0.13-1.34) | 0.142 | / | / |
| HRD status: medium *vs* low | 3.38(0.71-16.02) | 0.125 | / | / | 2.63(0.31-22.66) | 0.378 | / | / |
| HRD status: high *vs* low | 4.43(0.81-24.24) | 0.087 | / | / | 4.15(0.50-34.51) | 0.187 | / | / |

| **Variables** | **DFI** | | | | | | | |
| --- | --- | --- | --- | --- | --- | --- | --- | --- |
| Tumor size: T3-T4 *vs* T1-T2 | 1.78(0.60-5.23) | 0.296 | / | / | 2.06(0.60-7.10) | 0.252 | / | / |
| Lymph nodes: N2-N3 *vs* N0-N1 | 7.43(2.77-19.91) | <0.001 | 7.43(2.77-19.91) | <0.001 | 1.92(0.73-5.06) | 0.188 | / | / |
| HR status: Positive *vs* Negative | 0.43(0.14-1.34) | 0.147 | / | / | 0.36(0.15-0.90) | 0.029 | 0.36(0.15-0.90) | 0.029 |
| HRD status: medium *vs* low | 1.88(0.58-6.16) | 0.295 | / | / | 1.58(0.33-7.61) | 0.571 | / | / |
| HRD status: high *vs* low | 1.57(0.35-7.06) | 0.554 | / | / | 3.55(0.78-16.22) | 0.102 | / | / |

| **Variables** | **PFI** | | | | | | | |
| --- | --- | --- | --- | --- | --- | --- | --- | --- |
| Tumor size: T3-T4 *vs* T1-T2 | 1.06(0.49-2.28) | 0.883 | / | / | 2.33(0.93-8.81) | 0.071 | / | / |
| Lymph nodes: N2-N3 *vs* N0-N1 | 2.51(1.20-5.24) | 0.014 | 2.36(1.13-4.94) | 0.023 | 2.15(0.96-4.83) | 0.064 | / | / |
| HR status: Positive *vs* Negative | 0.54(0.24-1.24) | 0.144 | / | / | 0.42(0.19-0.92) | 0.030 | 0.42(0.19-0.92) | 0.030 |
| HRD status: medium *vs* low | 3.03(1.29-7.13) | 0.011 | 2.89(1.23-6.80) | 0.015 | 1.67(0.46-6.09) | 0.438 | / | / |
| HRD status: high *vs* low | 2.02(0.68-6.02) | 0.208 | / | / | 3.16(0.90-11.08) | 0.073 | / | / |

Abbreviation: DSS, Disease-specific Survival; DFI, Disease-free Interval; PFI, Progression-free Interval; HR, Hazard Ratio; HER2, Human epidermal growth factor receptor 2; IHC, Immunohistochemistry; HR, Hormone Receptor; HRD, Homologous Recombination Defect; HRRGs, Homologous Recombination Repair Genes; BRCA, Breast cancer susceptibility gene
