# Supplementary material for: Association between Homologous Recombination Repair Defect Status and Long-Term Prognosis of Early HER2-Low Breast Cancer: A Retrospective Cohort Study
Source: Oncologist. 2024 Feb 16;29(7):e864–76. doi: 10.1093/oncolo/oyae021 (PMC11224982; doi:10.1093/oncolo/oyae021)
Supplement: oyae021_suppl_Supplementary_Table_S5 [file oyae021_suppl_supplementary_table_s5.docx]

**Supplementary Table 5. Relationship of HRD and survival prognosis according to tumor size subgroups in TCGA-EBC**

| **Variables** | **T1-T2** | | | | **T3-T4** | | | |
| --- | --- | --- | --- | --- | --- | --- | --- | --- |
|  | Univariable analysis | | | | Multivariable analysis | | | |
|  | OR (95% CI) | *P* value | OR (95% CI) | *P* value | OR (95% CI) | *P* value | OR (95% CI) | *P* value |
|  | **DSS** | | | | | | | |
| Age: ≥60 *vs* <60 | 1.72(0.68-4.36) | 0.250 | / | / | 1.24(0.29-5.30) | 0.777 | / | / |
| Lymph nodes: N2-N3 *vs* N0-N1 | 3.89(1.51-10.08) | 0.005 | 3.98(1.54-10.28) | 0.004 | 0.75(0.15-3.77) | 0.727 | / | / |
| HR status: Positive *vs* Negative | 0.55(0.21-1.47) | 0.230 | / | / | 0.08(0.02-0.35) | 0.001 | 0.08(0.18-0.35) | 0.001 |
| HRD status: medium *vs* low | 2.38(0.51-11.27) | 0.273 | / | / | 5.75(0.66-50.04) | 0.113 | / | / |
| HRD status: high *vs* low | 3.75(0.80-17.65) | 0.095 | / | / | 6.89(0.60-78.80) | 0.121 | / | / |

| **Variables** | **DFI** | | | | | | | |
| --- | --- | --- | --- | --- | --- | --- | --- | --- |
| Age: ≥60 *vs* <60 | 1.13(0.53-2.44) | 0.750 | / | / | 1.01(0.24-4.32) | 0.991 | / | / |
| Lymph nodes: N2-N3 *vs* N0-N1 | 3.28(1.47-7.32) | 0.004 | 3.28(1.47-7.32) | 0.004 | 2.97(0.70-12.70) | 0.142 | / | / |
| HR status: Positive *vs* Negative | 0.49(0.22-1.08) | 0.077 | / | / | 0.05(0.01-0.24) | <0.001 | 0.05(0.01-0.24) | <0.001 |
| HRD status: medium *vs* low | 2.35(0.67-8.25) | 0.184 | / | / | 1.03(0.19-5.52) | 0.970 | / | / |
| HRD status: high *vs* low | 3.33(0.93-11.96) | 0.065 | / | / | 3.04(0.48-19.11) | 0.236 | / | / |

| **Variables** | **PFI** | | | | | | | |
| --- | --- | --- | --- | --- | --- | --- | --- | --- |
| Age: ≥60 *vs* <60 | 2.16(1.20-3.87) | 0.010 | 2.41(1.33-4.35) | 0.004 | 1.04(0.37-2.96) | 0.941 | / | / |
| Lymph nodes: N2-N3 *vs* N0-N1 | 2.43(1.28-4.62) | 0.007 | 2.80(1.46-5.38) | 0.002 | 1.19(0.42-3.43) | 0.744 | / | / |
| HR status: Positive *vs* Negative | 0.71(0.37-1.35) | 0.296 | / | / | 0.08(0.03-0.26) | <0.001 | 0.08(0.03-0.26) | <0.001 |
| HRD status: medium *vs* low | 2.34(0.96-5.71) | 0.063 | / | / | 2.06(0.59-7.21) | 0.256 | / | / |
| HRD status: high *vs* low | 2.30(0.89-5.92) | 0.086 | / | / | 3.59(0.88-14.69) | 0.075 | / | / |

Abbreviation: DSS, Disease-specific Survival; DFI, Disease-free Interval; PFI, Progression-free Interval; HR, Hazard Ratio; HER2, Human epidermal growth factor receptor 2; IHC, Immunohistochemistry; HR, Hormone Receptor; HRD, Homologous Recombination Defect; HRRGs, Homologous Recombination Repair Genes; BRCA, Breast cancer susceptibility gene.
