# Supplementary material for: Association between Homologous Recombination Repair Defect Status and Long-Term Prognosis of Early HER2-Low Breast Cancer: A Retrospective Cohort Study
Source: Oncologist. 2024 Feb 16;29(7):e864–76. doi: 10.1093/oncolo/oyae021 (PMC11224982; doi:10.1093/oncolo/oyae021)
Supplement: oyae021_suppl_Supplementary_Table_S6 [file oyae021_suppl_supplementary_table_s6.docx]

**Supplementary Table 6. Relationship of HRD and survival prognosis according to lymph nodes subgroups in TCGA-EBC**

| **Variables** | **N0-N1** | | | | **N2-N3** | | | |
| --- | --- | --- | --- | --- | --- | --- | --- | --- |
|  | Univariable analysis | | Multivariable analysis | | Univariable analysis | | Multivariable analysis | |
|  | OR (95% CI) | *P* value | OR (95% CI) | *P* value | OR (95% CI) | *P* value | OR (95% CI) | *P* value |
|  | **DSS** | | | | | | | |
| Age: ≥60 *vs* <60 | 1.48(0.57-3.86) | 0.421 | / | / | 2.68(0.70-10.31) | 0.152 | / | / |
| Tumor size: T3-T4 *vs* T1-T2 | 3.39(1.24-9.27) | 0.017 | 2.80(1.46-5.38) | 0.002 | 0.62(0.13-3.03) | 0.558 | / | / |
| HR status: Positive *vs* Negative | 0.59(0.21-1.69) | 0.329 | / | / | 0.128(0.03-0.52) | 0.004 | 0.128(0.03-0.52) | 0.004 |
| HRD status: medium *vs* low | 2.26(0.62-8.23) | 0.216 | / | / | 0.00(0.00-0.00) | 0.938 | / | / |
| HRD status: high *vs* low | 1.73(0.39-7.76) | 0.473 | / | / | 0.00(0.00-0.00) | 0.933 | / | / |

| Variables | **DFI** | | | | | | | |
| --- | --- | --- | --- | --- | --- | --- | --- | --- |
| Age: ≥60 *vs* <60 | 0.77(0.32-1.86) | 0.556 | / | / | 4.06(1.30-12.66) | 0.016 | 3.80(1.16-12.46) | 0.027 |
| Tumor size: T3-T4 *vs* T1-T2 | 1.25(0.36-4.31) | 0.726 | / | / | 1.55(0.50-4.77) | 0.447 | / | / |
| HR status: Positive *vs* Negative | 0.55(0.22-1.36) | 0.196 | / | / | 0.13(0.04-0.39) | <0.001 | 0.14(0.04-0.43) | 0.001 |
| HRD status: medium *vs* low | 3.94(0.88-17.65) | 0.073 | / | / | 0.41(0.10-1.65) | 0.208 | / | / |
| HRD status: high *vs* low | 4.50(0.93-21.73) | 0.061 | / | / | 0.78(0.21-2.87) | 0.706 | / | / |

| **Variables** | **PFI** | | | | | | | |
| --- | --- | --- | --- | --- | --- | --- | --- | --- |
| Age: ≥60 *vs* <60 | 1.86(1.00-3.46) | 0.048 | 2.17(1.16-4.05) | 0.015 | 2.76(1.08-7.04) | 0.034 | 2.44(0.93-6.35) | 0.069 |
| Tumor size: T3-T4 *vs* T1-T2 | 1.65(0.79-3.49) | 0.186 | / | / | 0.94(0.35-2.50) | 0.904 | / | / |
| HR status: Positive *vs* Negative | 0.72(0.36-1.44) | 0.355 | / | / | 0.16(0.06-0.42) | <0.001 | 0.18(0.07-0.47) | <0.001 |
| HRD status: medium *vs* low | 3.014(1.23-7.31) | 0.015 | 3.44(1.41-8.44) | 0.007 | 0.71(0.20-2.52) | 0.591 | / | / |
| HRD status: high *vs* low | 2.28(0.83-6.28) | 0.111 | 2.82(1.01-7.88) | 0.048 | 1.21(0.37-4.01) | 0.752 | / | / |

Abbreviation: DSS, Disease-specific Survival; DFI, Disease-free Interval; PFI, Progression-free Interval; HR, Hazard Ratio; HER2, Human epidermal growth factor receptor 2; IHC, Immunohistochemistry; HR, Hormone Receptor; HRD, Homologous Recombination Defect; HRRGs, Homologous Recombination Repair Genes; BRCA, Breast cancer susceptibility gene.
