# Supplementary material for: Association between Homologous Recombination Repair Defect Status and Long-Term Prognosis of Early HER2-Low Breast Cancer: A Retrospective Cohort Study
Source: Oncologist. 2024 Feb 16;29(7):e864–76. doi: 10.1093/oncolo/oyae021 (PMC11224982; doi:10.1093/oncolo/oyae021)
Supplement: oyae021_suppl_Supplementary_Table_S7 [file oyae021_suppl_supplementary_table_s7.docx]

**Supplementary Table 7. Relationship of HRD and survival prognosis according to HR status subgroups in TCGA-EBC**

| **Variables** | **HR-negative** | | | | **HR-positive** | | | |
| --- | --- | --- | --- | --- | --- | --- | --- | --- |
|  | Univariable analysis | | Multivariable analysis | | Univariable analysis | | Multivariable analysis | |
|  | OR (95% CI) | *P* value | OR (95% CI) | *P* value | OR (95% CI) | *P* value | OR (95% CI) | *P* value |
|  | **DSS** | | | | | | | |
| Age: ≥60 *vs* <60 | 1.92(0.51-7.16) | 0.334 | / | / | 1.82(0.69-4.80) | 0.227 | / | / |
| Tumor size: T3-T4 *vs* T1-T2 | 10.33(2.56-41.71) | 0.001 | 5.73(1.18-27.88) | 0.030 | 1.69(0.59-4.82) | 0.329 | / | / |
| Lymph nodes: N2-N3 *vs* N0-N1 | 6.16(1.65-23.05) | 0.007 | 3.60(0.81-15.92) | 0.092 | 1.77(0.62-5.02) | 0.286 | / | / |
| HRD status: medium *vs* low | 0.00(0.00-0.00) | 0.949 | / | / | 2.18(0.59-8.07) | 0.244 | / | / |
| HRD status: high *vs* low | 0.00(0.00-0.00) | 0.952 | / | / | 3.41(0.81-14.35) | 0.094 | / | / |

| **Variables** | **DFI** | | | | | | | |
| --- | --- | --- | --- | --- | --- | --- | --- | --- |
| Age: ≥60 *vs* <60 | 1.25(0.38-4.07) | 0.712 | / | / | 1.49(0.64-3.47) | 0.353 | / | / |
| Tumor size: T3-T4 *vs* T1-T2 | 14.70(3.83-56.37) | <0.001 | 3.36(0.65-17.43) | 0.149 | 1.65(0.60-4.55) | 0.332 | / | / |
| Lymph nodes: N2-N3 *vs* N0-N1 | 9.03(2.99-27.28) | <0.001 | 5.91(1.51-23.13) | 0.011 | 2.67(1.12-6.38) | 0.027 | 2.67(1.12-6.38) | 0.027 |
| HRD status: medium *vs* low | 0.29(0.05-1.62) | 0.160 | / | / | 1.95(0.63-6.08) | 0.247 | / | / |
| HRD status: high *vs* low | 0.22(0.05-1.08) | 0.062 | / | / | 3.26(0.92-11.59) | 0.068 | / | / |

| **Variables** | **PFI** | | | | | | | |
| --- | --- | --- | --- | --- | --- | --- | --- | --- |
| Age: ≥60 *vs* <60 | 1.74(0.67-4.51) | 0.256 | / | / | 2.27(1.21-4.25) | 0.011 | 2.64(1.40-4.98) | 0.003 |
| Tumor size: T3-T4 *vs* T1-T2 | 10.38(3.58-30.10) | <0.001 | 5.03(1.40-18.09) | 0.013 | 1.20(0.59-2.45) | 0.618 | / | / |
| Lymph nodes: N2-N3 *vs* N0-N1 | 6.14(2.34-16.08) | <0.001 | 3.42(1.06-10.96) | 0.039 | 1.67(0.86-3.26) | 0.130 | / | / |
| HRD status: medium *vs* low | 0.38(0.07-1.98) | 0.253 | / | / | 2.48(1.12-5.47) | 0.024 | 2.88(1.30-6.40) | 0.009 |
| HRD status: high *vs* low | 0.35(0.08-1.60) | 0.177 | / | / | 2.28(0.85-6.08) | 0.101 | 2.96(1.09-8.00) | 0.033 |

Abbreviation: DSS, Disease-specific Survival; DFI, Disease-free Interval; PFI, Progression-free Interval; HR, Hazard Ratio; HER2, Human epidermal growth factor receptor 2; IHC, Immunohistochemistry; HR, Hormone Receptor; HRD, Homologous Recombination Defect; HRRGs, Homologous Recombination Repair Genes; BRCA, Breast cancer susceptibility gene
