# Supplementary material for: Association between Homologous Recombination Repair Defect Status and Long-Term Prognosis of Early HER2-Low Breast Cancer: A Retrospective Cohort Study
Source: Oncologist. 2024 Feb 16;29(7):e864–76. doi: 10.1093/oncolo/oyae021 (PMC11224982; doi:10.1093/oncolo/oyae021)
Supplement: oyae021_suppl_Supplementary_Figure_Captions [file oyae021_suppl_supplementary_figure_captions.docx]

**Supplementary Figure Captions**

**Supplementary Figure 1:** Process for screening enrolled patients.

**Supplementary Figure 2:** The molecular landscape of the TCGA and SYSU HER2-low breast cancer cohort.

**Supplementary Figure 3:** TCGA HER2‑low early breast cancers immunological tumor landscape in XCELL and CIBERSORT-ABS algorithms according to HRD status.

**Supplementary Figure 4(A-F):** Kaplan-Meier curve analysis of HER2-low TCGA-EBC patients with different HRD status and long-term survival in in HR and Lymph nodes subtype

**Supplementary Figure 5:** Kaplan-Meier curve analysis of HER2-0 patients with different HRD status and long-term survival in TCGA-EBC cohorts.

**Supplementary Figure 6:** Results of HRD status in subgroup analysis at DSS of HER2-low patients in TCGA-EBC cohorts.

**Supplementary Figure 7:** Results of HRD status in subgroup analysis at DFI of HER2-low patients in TCGA-EBC cohorts.
